# Competitive adsorption of CO_2_, N_2_, and CH_4_ in coal-derived asphaltenes, a computational study

**DOI:** 10.1038/s41598-024-58347-6

**Published:** 2024-04-01

**Authors:** Farshad Mirzaee Valadi, Mohammad Pasandideh-Nadamani, Mozafar Rezaee, Abdolhalim Torrik, Mohammad Mirzaie, Ayoob Torkian

**Affiliations:** 1 Water and Energy Research Center, Sharif University of Technology, Tehran, Iran; 2 Department of Chemistry, University of Mazanderan, Babolsar, Mazanderan Iran; 3 Department of Chemistry, Iran University of Science and Technology, Tehran, Iran; 4 Department of Physical and Computational Chemistry, Shahid Beheshti University, Tehran, Iran

**Keywords:** Energy, Environmental chemistry, Physical chemistry, Surface chemistry, Theoretical chemistry

## Abstract

Greenhouse gases are major contributors to global warming, and their concentration is increasing due to the widespread use of fossil fuels. Coal bed methane (CBM) offers a potential solution to this issue. However, the gas adsorption mechanisms of CBM, particularly in the context of coal-derived asphaltenes, are not fully understood. This study provides a comprehensive theoretical investigation of the competitive adsorption of carbon dioxide (CO$_2$), methane (CH$_4$), and nitrogen (N$_2$) in the processes of CO$_2$- and N$_2$-enhanced coalbed methane recovery, with a focus on coal-derived asphaltenes functionalized with CH$_4$, NH, O, and S groups. Using the Grand Canonical Monte Carlo (GCMC) simulation method and performing Molecular Dynamics (MD) simulations, we studied the adsorption process. To investigate the electronic effects and nature of the interactions, we performed density functional theory (DFT) calculations. The adsorption energy values and non-covalent interactions (NCI) for the adsorption of gases signify the physical adsorption (van der Waals interaction), with CO$_2$ exhibiting the highest (absolute) adsorption energy. The Monte Carlo results indicated that elevated temperatures led to a reduction in adsorption capacity. Coal-derived asphaltenes demonstrated greater selectivity for CO$_2$ compared to CH$_4$ and N$_2$ in competitive adsorption, especially at elevated temperatures. Our findings highlight the significant potential of our asphaltene model, not only in mitigating CO$_2$ greenhouse gas emissions but also in recovering CH$_4$, which is a valuable resource.

## Introduction

Greenhouse gases are a major contributor to the increase in Earth’s temperature. This increase can lead to destructive changes on a global scale, such as the melting of natural glaciers, rising sea levels, tropical storms, climate change, and ocean acidification. As such, the Kyoto Protocol was established in 1997 with the aim of reducing greenhouse gas emissions. This protocol introduces six greenhouse gases: CO$_2$, CH$_4$, nitrous oxide, HFCs, PFCs, and sulfur hexafluoride. Among these gases, CO$_2$ is the most concerning because it is the most abundant of the six gases and has the largest contribution to climate change and global warming. China is the largest energy consumer and produces the highest amount of CO$_2$ globally, with a 30% share of the total emissions^1,2^. It is widely acknowledged that CO$_2$ concentrations are continuously increasing in the atmosphere, primarily due to the use of fossil fuels for energy generation. Currently, fossil fuels supply over 80% of the world’s commercial energy^3,4^. A potent approach to combat climate change and global warming is to utilize coal bed methane (CBM)^5,6^. Coal bed methane (CBM), a significant source of unconventional natural gas worldwide, mainly consists of substantial amounts of CH$_4$, minimal quantities of CO$_2$ and N$_2$, and trace amounts of other gases^7,8^. Numerous techniques have been employed to enhance CBM extraction, and gas injection is one of the most important, straightforward, and cost-effective approaches^9,10^. Gas injection is a valuable method for enhancing CH$_4$ recovery, while also providing a practical solution for CH$_4$ disposal and maintaining tank pressure. In a gas-injection-enhanced coalbed methane recovery project, gas is injected into deep, unextractable coal seams, and the resulting CH$_4$ is recovered for use as fuel or clean gas^11,12^. CO$_2$ and N$_2$ are commonly selected as injection gases to enhance CBM recovery because of their favorable structural characteristics for competitive adsorption with CH$_4$ as well as their easy accessibility^11,13^. This strategy offers numerous benefits, such as preventing mine explosions, enhancing CH$_4$ recovery and utilization, utilizing abundant resources, lowering atmospheric CO$_2$ concentration, mitigating global warming, and achieving high efficiency. Therefore, CBM mining has become a popular topic^8,11^. In the process of adsorption for CH$_4$ recovery in the coal bed, N$_2$ (N$_2$-ECBM) and CO$_2$ (CO$_2$-ECBM) molecules compete with CH$_4$ for adsorption sites, which forms the theoretical basis for methane recovery in the coal bed^14^. Therefore, it is important to gain a deep insight into the adsorbent structure and adsorption mechanism during the competitive adsorption process. Coal-derived asphaltenes, distinguished by their dense, active polyaromatic structure and distinctive characteristics, play a pivotal role in adsorbing gases in both the CO$_2$-Enhanced Coalbed Methane Recovery (CO$_2$-ECBM) and N$_2$-Enhanced Coalbed Methane Recovery (N$_2$-ECBM) processes. Notably, heteroatoms such as N, O, and S can be found within asphaltene molecules, contributing to their remarkable properties^15,16^.

Recently, there has been a growing interest in applying computational methods such as MD, DFT, and GCMC to study and analyze competitive adsorption on asphaltene structures derived from coal^11,17^. In a previous study conducted by one of the authors^18^, theoretical GCMC, MD, and DFT methods were employed to examine the impact of the pyrrole functional group in asphaltene derived from model coal on the selective adsorption of CO$_2$. These research findings indicate that the NH functional group enhances the selective adsorption of CO$_2$. In this study, we utilized the MD, DFT, and GCMC methods to investigate the competitive adsorption of CO$_2$, N$_2$, and CH$_4$ in the CO$_2$-ECBM and N$_2$-ECBM processes on a model asphaltene doped with CH$_2$, NH, O, and S atoms.

## Method

### Molecular model

**Figure 1:**
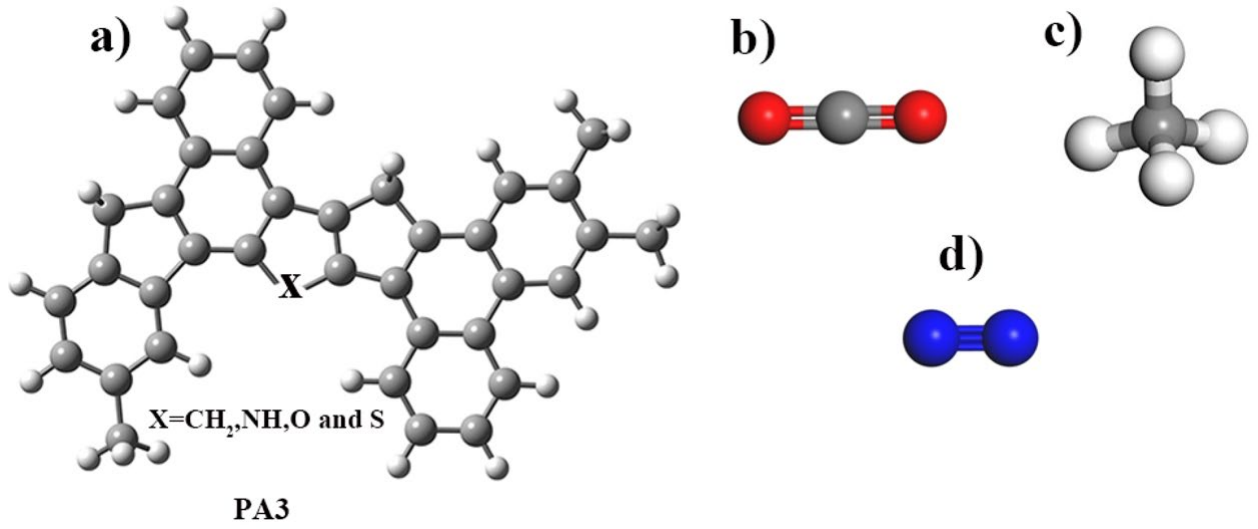
Molecular structures of (**a**) PA3, (**b**) CO$_2$, (**c**) CH$_4$, and (**d**) N$_2$.

For this research, we referred to the study conducted by Schuler et al. to select an asphaltene molecule^19^. Their work utilized both atomic force microscopy (AFM) and scanning tunneling microscopy (STM) to reliably identify asphaltenes, thereby establishing a robust foundation for structure identification. Therefore, we chose PA3 as the adsorbent. Molecular structures of the asphaltene model and adsorbate gases are shown in Fig. 1. According to reference articles^19,20^, in position X (see Fig. 1), there can be a heteroatom such as CH$_2$, NH, or O. For the purpose of this study, we incorporated CH$_2$, NH, O, and S groups as heteroatoms into the asphaltene structure to assess the adsorption of CO$_2$, CH$_4$, and N$_2$.

### Grand canonical Monte Carlo and molecular dynamics

**Figure 2:**
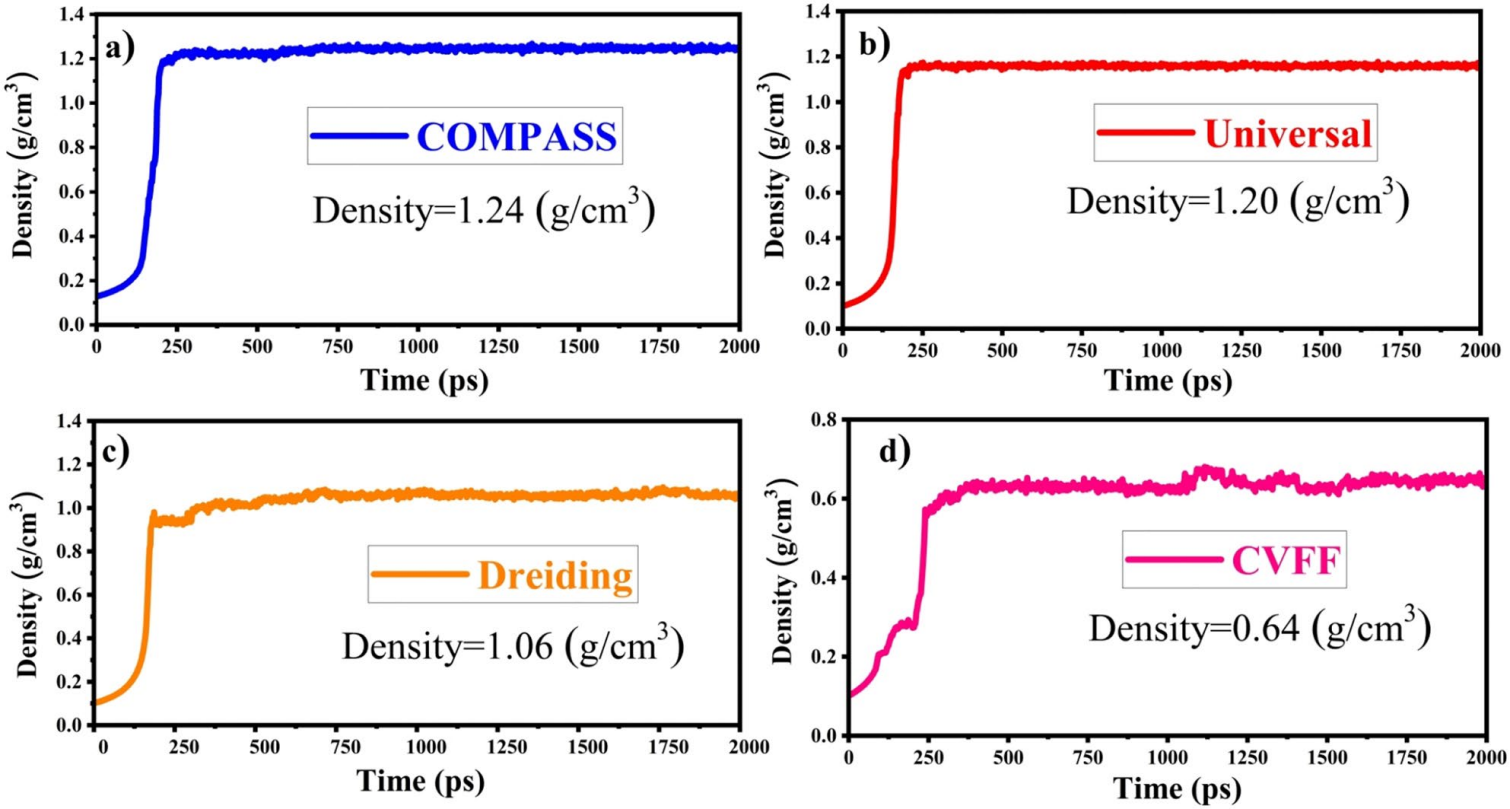
Density in the dynamics process. The NVT ensemble system was thermalized and equilibrated using 200 ps at 298 K. The NPT ensemble was used for 2 ns. The first 1 ns were used for relaxation, and the last 1 ns were used to calculate the density. The Nosé-Hoover thermostat and Berendsen barostat were used to control the temperature and pressure, respectively.

An appropriate force field is crucial for molecular simulations to accurately represent atomic interactions and align with experimental outcomes. In our research on coal products, we evaluated various force fields, including Dreiding, CVFF, PCFF, and COMPASS, based on a literature review^21–23^. We compared the molecular densities of PA3 obtained from different force fields with the experimentally determined actual densities of coal products. The results, detailed in Fig. 2, indicate that the COMPASS force field closely approximates real values.

**Figure 3:**
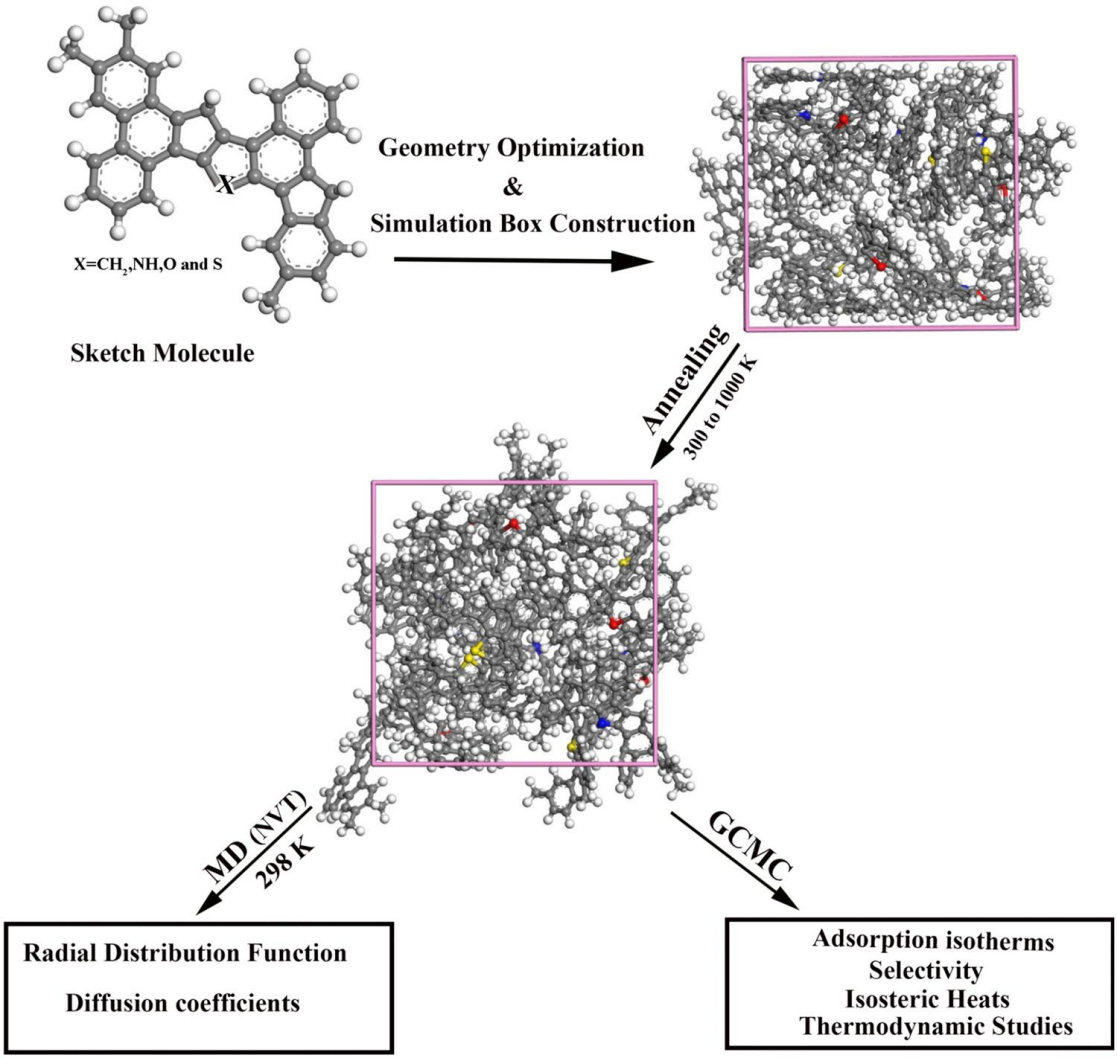
The process of molecular simulation for PA3.

In addition, the COMPASS force field has a proven track record for simulating oil molecules and CO$_2$ adsorption^22,24^. Consequently, for all simulations, COMPASS was adopted as the force field, with a cutoff radius of 12.5 angstroms. Furthermore, we assessed the adsorption isotherms obtained through our simulations and experimental data^25^, as presented in Fig. S1, to validate our methodology. Both datasets showed strong agreement with the Langmuir–Freundlich model.

Initially, we constructed a simulation container with 20 PA3 molecules (comprising five types: PA3C, PA3O, PA3N, and PA3S), in which the density was 1.2 g/cm$^3$. We utilized the SMART minimization algorithm to perform geometry optimization and obtain the initial configuration^26,27^. Using the Amorphous Cell module of the Materials Studio software, we generated coal-derived asphaltene. Subsequently, we conducted equilibration through annealing dynamics at various temperatures (300–1000 K), as illustrated in Fig. 3.

We utilized the GCMC technique with the Metropolis algorithm, employing the sorption module within Materials Studio, to investigate the selective adsorption of gases and establish the adsorption isotherms^28^. The temperature range of the simulations was 273–373 K, while the pressure was fixed (0–10000 kPa). The simulation parameters were chosen as $10^6$ equilibration steps and $10^7$ production steps. The van der Waals interactions were examined using the Lennard-Jones potential, while the electrostatic interactions were investigated using the Coulombic term. For the MD simulations, the NVT ensemble with a Nose–Hoover temperature thermostat was used. The analysis focused on the final 0.4 ns of each MD simulation, which covered a total duration of 2 ns at 298 K, with a time step of 1 fs.

### Density functional theory

In this study, we utilized DFT calculations to investigate the impact of substituting four heteroatoms with each other on the electronic properties of asphaltene molecules derived from coal. Our aim was to enhance our comprehension of the adsorption process of CH$_4$, N$_2$, and CO$_2$ molecules on asphaltene. DFT calculations were performed using Gaussian 09 software based on simulations from previous studies^29–31^. Analyzes such as theoretical reactivity parameters (hardness, chemical potential, and electrophilicity) and ESP maps were obtained using the B3LYP exchange-correlation functional and 6-311++G(d,p) basis set. We chose the B3LYP exchange-correlation functional for computing electronic parameters because of its accuracy and low computational cost. We optimized the PA3, gas molecules, and PA3-gas molecule complex with the $\omega$B97XD exchange-correlation functional and 6-31+G(d,p) basis set. The adsorption energy was calculated using this level of theory. The reason we chose this exchange-correlation function was that it considers dispersion interactions due to its unique formalism^32,33^. We conducted a non-covalent interaction (NCI) analysis using the optimized adsorbent-adsorbate geometry at the $\omega$B97XD/6-31+G(d,p) level of theory. Furthermore, we performed a non-covalent interaction (NCI) analysis using Multiwfn and VMD software.

## Results and discussion

### Monte Carlo in the grand canonical ensemble

#### Adsorption competition

Adsorption isotherms are essential tools for establishing a relationship between adsorption loading and different pressures at a constant temperature. This study focuses on examining the adsorption tendencies of CO$_2$, CH$_4$, and N$_2$ within a model coal-derived asphaltene using the GCMC method. This investigation was conducted across a temperature spectrum, including 273 K, 298 K, 323 K, and 348 K, while the pressure ranged from 0.0-10000 kPa. In the following, we introduce the models used to describe the adsorption process.

The Langmuir model^34^ is based on the notion that adsorbate molecules adhere to the surface of an adsorbent to form a single molecular layer. This model assumes that the surface of the adsorbent features uniform binding sites^35^. The Langmuir model is represented by the following equation^34^:

1 $$\begin{aligned} q=\frac{abp}{1+abp} \end{aligned}$$

Parameter $a$ signifies the monolayer adsorption capacity (mL/g), $b$ corresponds to the adsorption equilibrium constant (kPa$^{-1}$), $p$ is the pressure (kPa), and $q$ represents the adsorption amount in units of (mL/g).

Furthermore, the Freundlich model was used to analyze the adsorption isotherms. This empirical isotherm was designed to represent multilayer (heterogeneous) adsorption. The mathematical representation of the Freundlich model is given by the following equation^34^:

2 $$\begin{aligned} q = kp^{\frac{1}{n}} \end{aligned}$$

here, *k* is Freundlich constant (mLg$^{-1}$kPa$^{-\frac{1}{n}}$), and *n* characterizes the heterogeneity of the adsorbent. In the context of adsorption, an *n* value greater than one indicates that the adsorbate is more likely to bind to the adsorbent surface.

Although both previously described models find utility across various systems, their individual limitations have motivated the adoption of a combined approach. This is a result of the inherent shortcomings of both models^36^. The integration of these equations results in the Langmuir–Freundlich model, which effectively addresses the intrinsic heterogeneity of adsorbents. The following equation encapsulates this model^11^:

3 $$\begin{aligned} q = \frac{abp^{\frac{1}{n}}}{1+bp^{\frac{1}{n}}} \end{aligned}$$

The Langmuir–Freundlich model effectively represents numerous heterogeneous systems^37,38^. In this model, variables *a* and *b* demonstrate a significant inverse relationship with the temperature^11,39^. Parameter $b$ quantifies the inclination of the gas to adhere to the surface of an adsorbent^40^, whereas $n$ indicates the heterogeneity of the adsorbent surface.

**Figure 4:**
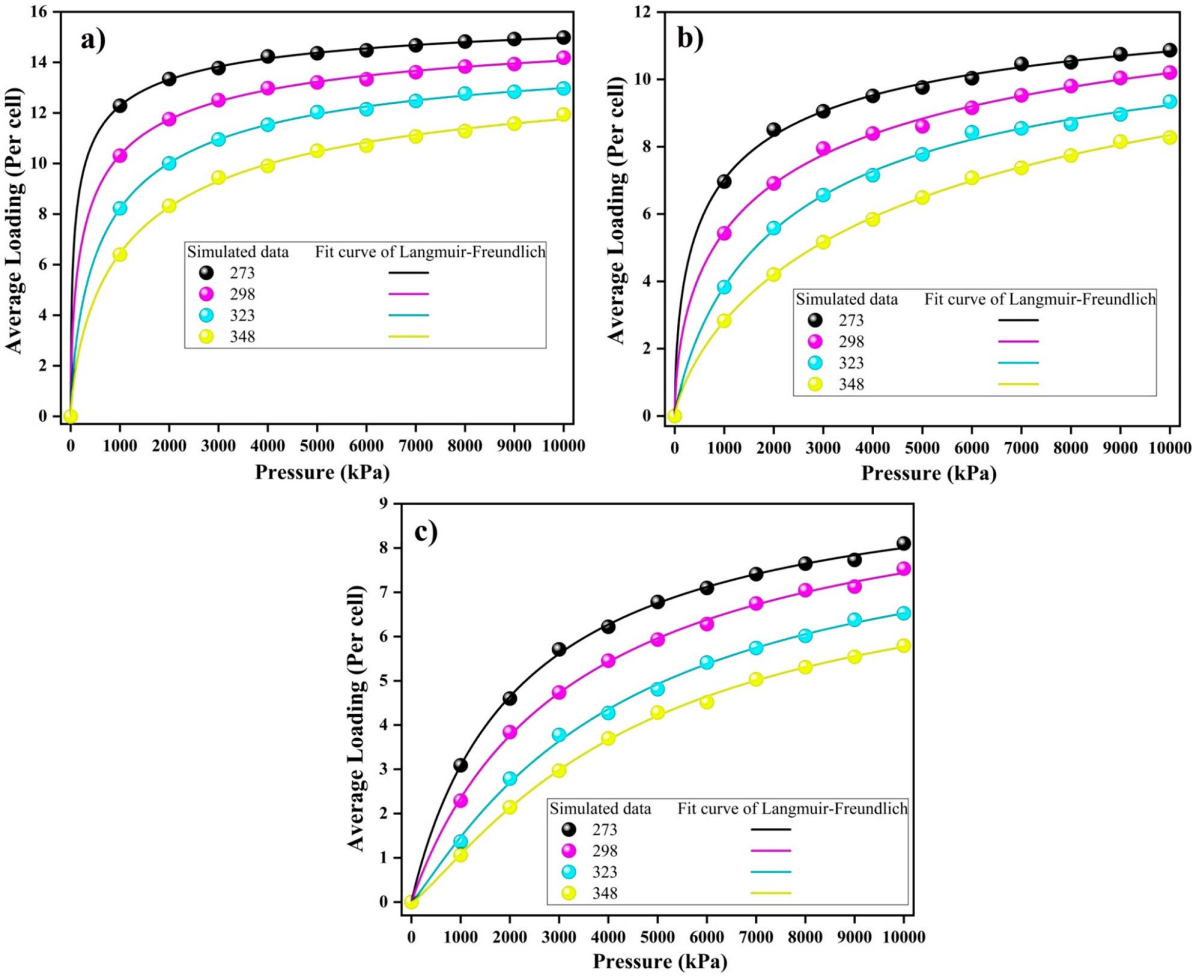
Langmuir–Freundlich isotherms for (**a**) CO$_2$, (**b**) CH$_4$, and (**c**) N$_2$ on model coal-derived asphaltene at various temperatures.

Figure 4 illustrates the changes in adsorption capacity in response to pressure variations for CO$_2$, CH$_4$, and N$_2$ at different temperatures based on the Langmuir–Freundlich isotherm (refer to Figs. S2, S3 for the Freundlich and Langmuir isotherms, respectively). The data indicate a clear increase in the adsorption capacity for each gas as the pressure increases. As expected, the adsorption capacities of all gases were negatively correlated with changes in temperature^40^. Furthermore, heterogeneous adsorption on the surface of PA3 is evident, as the adsorption rate decreases with increasing pressure. Initially, gas molecules preferentially occupy available sites, and as pressure increases, sites with varying energy levels become occupied. Based on this figure, the average load per cell at 273 K is as follows:

CO$$_2$$ (14.20) > CH$$_4$$ (10.20) > N$$_2$$ (7.60).

Clearly, the contrast in the adsorption quantity is more pronounced for CO$_2$ than for CH$_4$ and N$_2$. The reasons for these results are thoroughly explored in the Adsorption Energy Calculation section. Furthermore, Table 1 presents the relevant parameters and $R^2$ values for the gases, as determined by the Langmuir–Freundlich model. Additional findings derived from both the Langmuir and Freundlich models are presented in the Supporting Information, especially in Tables S2–S4. Remarkably, the model effectively encapsulated the adsorption patterns exhibited by all gases on the model coal-derived asphaltene, as indicated by the $R^2$ values.

**Table 1:** Langmuir–Freundlich model coefficients for adsorption isotherms of CO$_2$, CH$_4$, and N$_2$ on model coal-derived asphaltene at various temperatures.

Adsorbent	T(K)	a (mL/g)	b (kPa$^{-1}$)	n	$R^{2*}$
CO$_2$	273	16.53	0.0791	1.91	0.9998
	298	16.09	0.0302	1.70	0.9997
	323	14.93	0.0075	1.35	0.9998
	348	14.43	0.0056	1.36	0.9991
CH$_4$	273	13.55	0.0211	1.76	0.9992
	298	12.91	0.0162	1.52	0.9989
	323	11.93	0.0014	1.34	0.9986
	348	11.12	0.0010	1.35	0.9996
N$_2$	273	9.94	0.0058	1.905	0.9992
	298	9.45	0.0036	1.366	0.9991
	323	8.96	0.0034	1.256	0.9982
	348	8.06	0.0026	1.143	0.9986

* Note: $R^2$: the nonlinear regression coefficient.

#### Selectivity of adsorption

**Figure 5:**
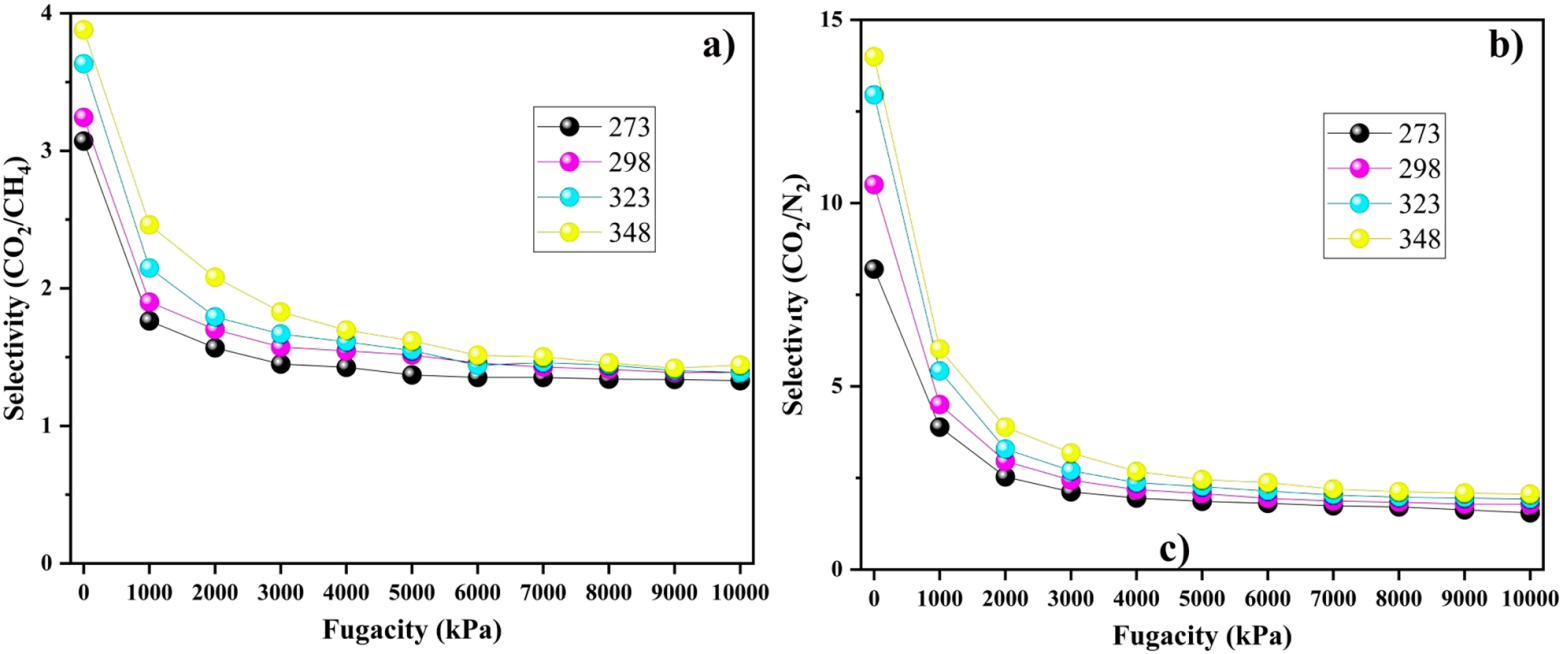
Selectivity of model coal-derived asphaltene for the adsorption of CO$_2$ over (**a**) CH$_4$ and (**b**) N$_2$ at different temperatures.

During adsorption, the selectivity parameter measures the relative affinity of an adsorbent for different adsorbates. We used it to assess the adsorption of CO$_2$, CH$_4$, and N$_2$ within PA3 in a competitive manner. The following equation defines the selectivity parameter^1^:

4 $$\begin{aligned} S = (\frac{x_{CO_{2}}}{x_{b}})/(\frac{y_{CO_{2}}}{y_{b}}), \hspace{10mm} b = \textrm{CH}_4, \textrm{CO}_2 \text {and} \textrm{N}_2 \end{aligned}$$

here, $x_i$ and $y_i$ denote the proportion of the gas component *i* in the adsorbed and bulk phases, respectively. Considering the distinct adsorption strengths of gases onto PA3, we conducted simulations at five different temperatures with varying pressures to investigate selectivity, see Fig. 5. From the figure, PA3 shows a higher selectivity for CO$_2$ than for N$_2$ and CH$_4$ at all temperatures. Furthermore, the selectivity increased as the temperature rose, indicating a positive correlation. When it comes to pressure, it exhibits a negative correlation with selectivity. At a certain threshold of high pressure, it counteracts the impact of temperature, resulting in a consistent selectivity value across all temperatures. This phenomenon arises because at low pressures (corresponding to low adsorption loading), more active sites are available. As the loading increased, the number of active sites decreased. Consequently, additional gas molecules must adhere to and occupy less favorable sites, resulting in reduced selectivity as the pressure increases^41^. Overall, due to the elevated selectivity of PA3 for CO$_2$, demonstrates greater adsorption capabilities than CH$_4$ and N$_2$.

#### Isosteric heats

To delve deeper into the selective adsorption of gases, we introduce the concept of the isosteric heat of adsorption^1^. Given that adsorption is inherently an exothermic process, evaluating the quantified heat release can offer insights into binding strength^11^. The isosteric heat $Q_{st}$ (Kcal/mol) was calculated using^14^:

5 $$\begin{aligned} Q_{\text {st}} = R_g T - \frac{\langle N_{\text {ad}} U_{\text {ad}} \rangle - \langle N_{\text {ad}} \rangle \langle U_{\text {ad}} \rangle }{\langle N^2_{\text {ad}} \rangle - \langle N_{\text {ad}} \rangle ^2} \end{aligned}$$

where T (K) is the temperature, $R_g$ (Kcal.mol$^{-1}.$K$^{-1}$) is the universal gas constant, $U_{ad}$ (Kcal) represents the energy of adsorption, and $N_{ad}$ (mol) denotes the total number of adsorbates.

**Figure 6:**
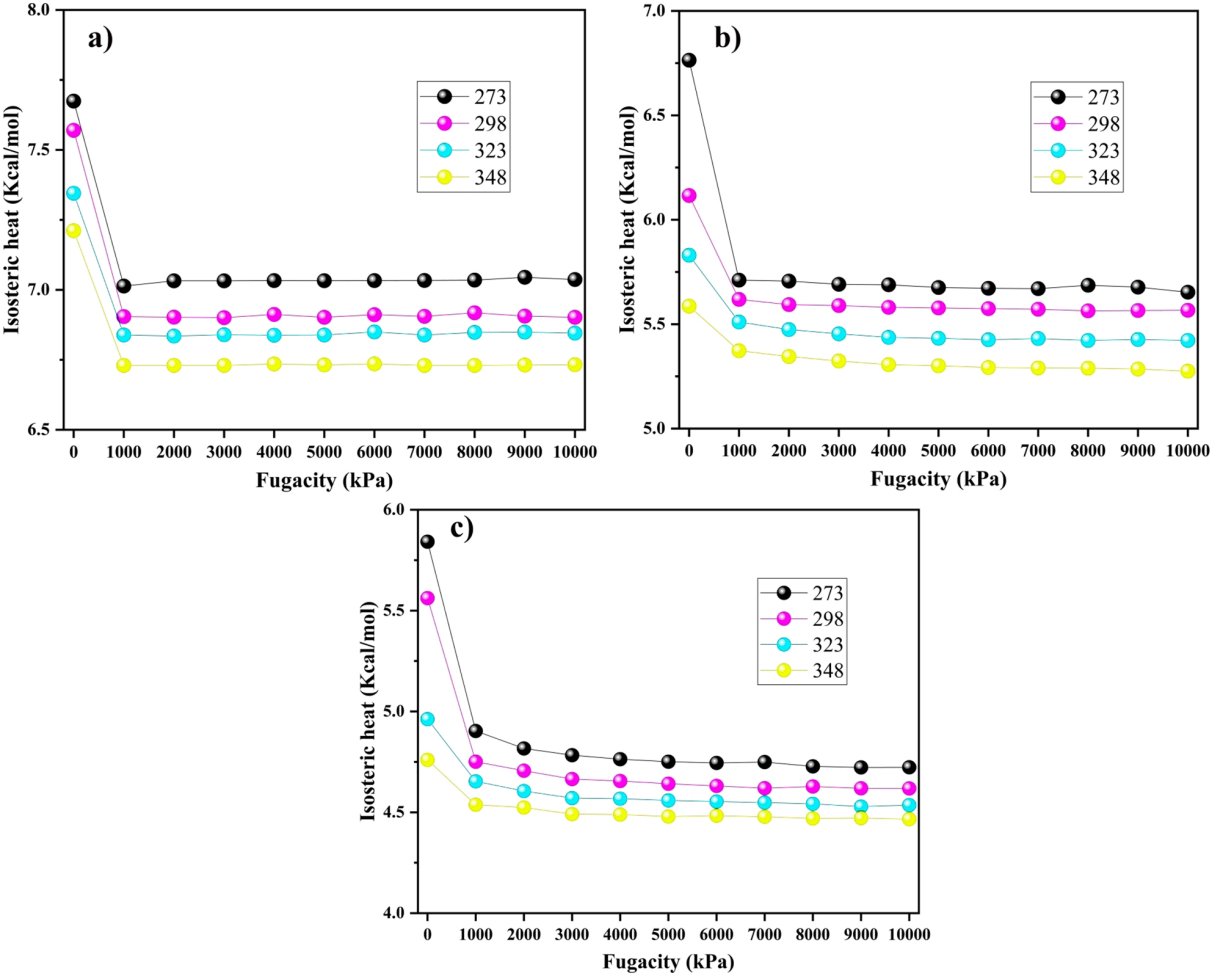
Isosteric heats of (**a**) CO$_2$, (**b**) CH$_4$, and (**c**) N$_2$ with varying adsorption pressure at different temperatures.

The corresponding $Q_{st}$ values for the adsorption of gases under varying pressure are illustrated in Fig. 6. Upon initial inspection of the plots, we observed a decrease in the isosteric heat with increasing temperature and pressure. The decrease in isosteric heat of adsorption with increasing temperature can be attributed to the exothermic nature of the process^42^. As the temperature increased, the thermal energy also increased, leading to a reduction in the energy gap between the adsorbed and non-adsorbed states. This weakens the binding strength, leading to lower isosteric heat. Moreover, isosteric heat is associated with the enthalpy change^43^. As the temperature increases, adsorption becomes less spontaneous, resulting in lower absolute values of the enthalpy change, and a decrease in isosteric heat.

The noticeable heat release observed at lower pressure levels may stem from the diverse energy levels of the active adsorption sites and the tendency of gas molecules to adhere to these sites, resulting in a significant generation of heat^8^. Subsequently, in the range of 0-10 MPa, due to the decrease in favorable sites, the isosteric heat curves for all gases stabilized. It should be noted that CO$_2$ has a higher isosteric heat than CH$_4$ and N$_2$, while being less sensitive to temperature. Furthermore, all gases showed isosteric heat values below 10 kcal/mol for adsorption on PA3, indicating a physisorption process^44,45^.

#### Assessment of adsorption affinities

To investigate the interaction of gases with the surface of the adsorbent, we utilized Henry’s constant^46^. Henry’s law describes a direct correlation between the amount of adsorption and the equilibrium pressure in the low-pressure regime. This relationship is expressed as follows^46^:

6 $$\begin{aligned} \frac{p}{q}&= \frac{1}{K_H}exp(A_1q+A_2q^2+A_3q^3+...) \end{aligned}$$

7 $$\begin{aligned} q&= K_Hp \end{aligned}$$

**Figure 7:**
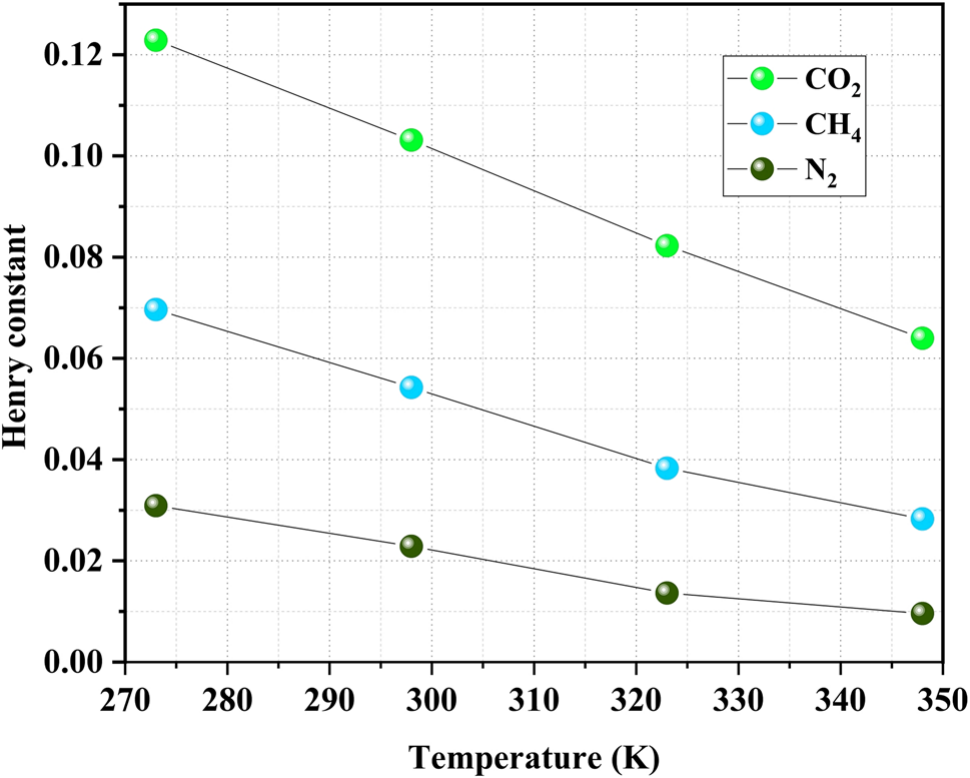
Henry constants of CO$_2$, CH$_4$, and N$_2$ in model coal-derived asphaltene at various temperatures.

In this equation, coefficients A$_1$, A$_2$, and A$_3$, along with Henry’s coefficient $K_H$ (mLg$^{-1}$kPa$^{-1}$), adsorption quantity $q$, and $p$ representing the equilibrium pressure, play key roles. A higher $K_H$ indicates a greater affinity between the adsorbate and adsorbent. $K_H$ values for the gases in the adsorption process at different temperatures are illustrated in Fig. 7. It is evident that the temperature correlates negatively with $K_H$; higher temperatures and $K_H$ significantly diminish the adsorption affinity of all gases. Compared to N$_2$ and CH$_4$, the Henry’s constant for CO$_2$ exhibits a slightly greater sensitivity to temperature changes. The variation in $K_H$ values for CO$_2$, CH$_4$, and N$_2$ reflects their effectiveness in the adsorption process. Notably, CO$_2$ exhibits a higher $K_H$ value than the other gases. Furthermore, the polarizabilities of CO$_2$, CH$_4$, and N$_2$ are 29.1, 25.9, and 17.6, respectively^47,48^. Considering these values, the increased polarizability of CO$_2$ makes its interaction with the surface of PA3 more effective than that of the other gases.

#### Thermodynamic analysis

Due to the crucial role of thermodynamic studies in understanding adsorption behavior, we conducted an in-depth analysis to gain a comprehensive understanding.

**Surface potential**

The surface potential energy $\Omega$ (J/g) represents the energy required for the adsorbate to detach from the adsorbent surface. We computed this using the following equation^11^:

8 $$\begin{aligned} \Omega = -RT \int _{0}^{p} \frac{q}{p}dp \end{aligned}$$

The universal gas constant is represented by R, the absolute temperature is denoted by T, and the adsorption loading is indicated by q. Molecules with a higher absolute surface potential exhibit greater adsorption onto the adsorbent surface^46^. Because adsorption is a process that releases energy, $\Omega$ takes values below zero.

**Figure 8:**
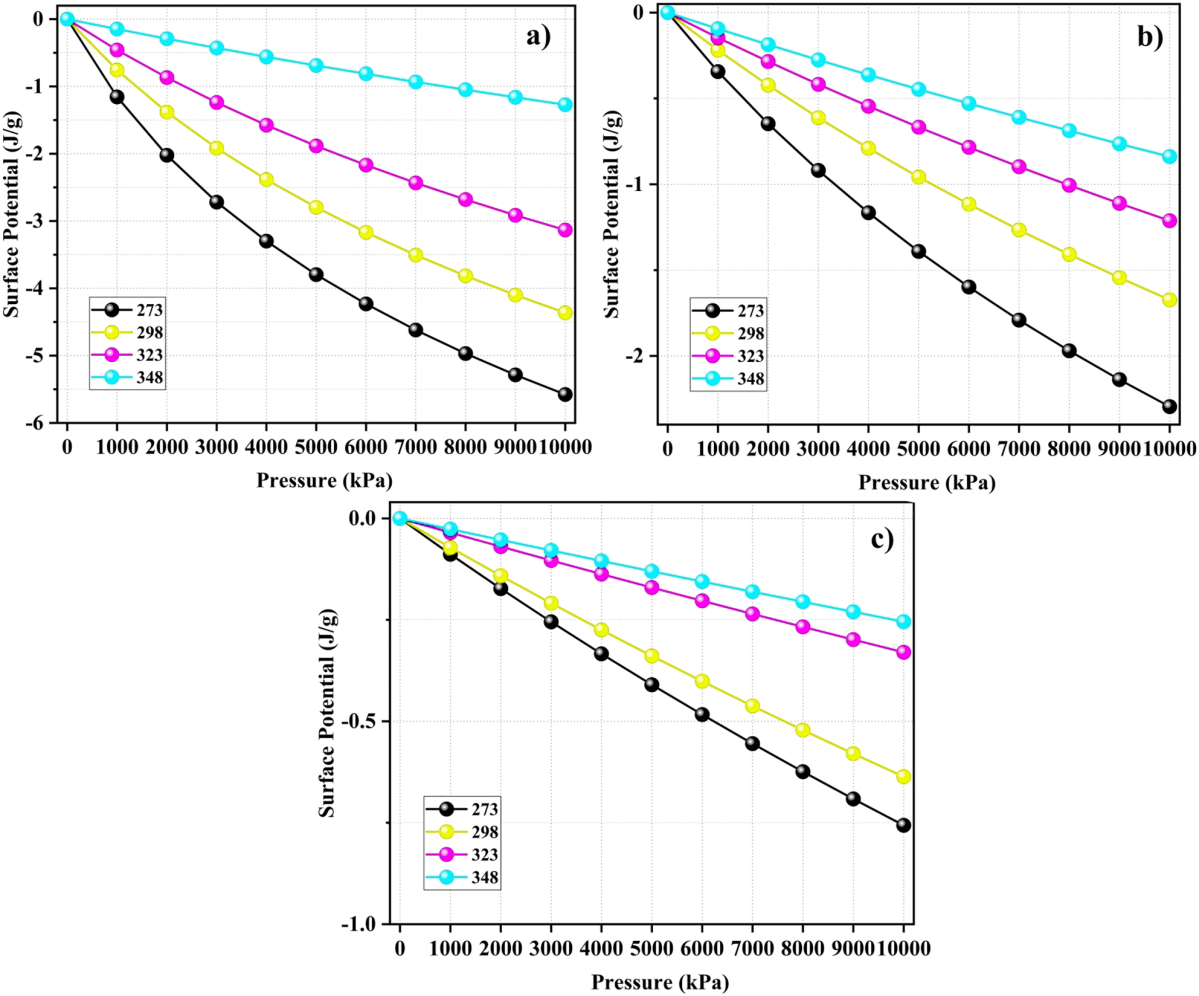
Surface potentials of (**a**) CO$_2$, (**b**) CH$_4$, and (**c**) N$_2$ in model coal-derived asphaltene at various temperatures.

Plots of $\Omega$ with varying pressures for the adsorption of CO$_2$, CH$_4$, and N$_2$ on PA3 are depicted in Fig. 8. For each simulation, the absolute magnitudes of the surface potential for the gases increased under elevated pressures. This phenomenon may arise from the need for additional energy to accommodate adsorbates within the cavities of the porous material under high-pressure conditions^49^. Furthermore, an increase in the absolute $\Omega$ values was observed for all gases when the temperature decreased. This contributed to the enhanced adsorption of gases on the surfaces of the adsorbents. Notably, for the adsorption of CO$_2$, the $\Omega$ value exhibited a significant increase with a decrease in temperature compared to the values of other gases, highlighting CO$_2$’s advantage in competitive adsorption on the surfaces of the adsorbents.

**Gibbs free energy**

The following formula was used to determine the change in Gibbs free energy $\Delta G$ (J/ml)^11^:

9 $$\begin{aligned} \Delta G = \frac{\Omega }{q} \end{aligned}$$

Following the principle of minimum energy, interfaces naturally tend to minimize their surface energy. For a solid interface, the minimization of surface energy occurs through the adsorption of other molecules onto its surface. This means that significant changes in the surface free energy result in pronounced adsorption loading^49^. The Gibbs Free Energy difference ($\Delta G$) indicates whether the adsorption process occurred spontaneously.

**Figure 9:**
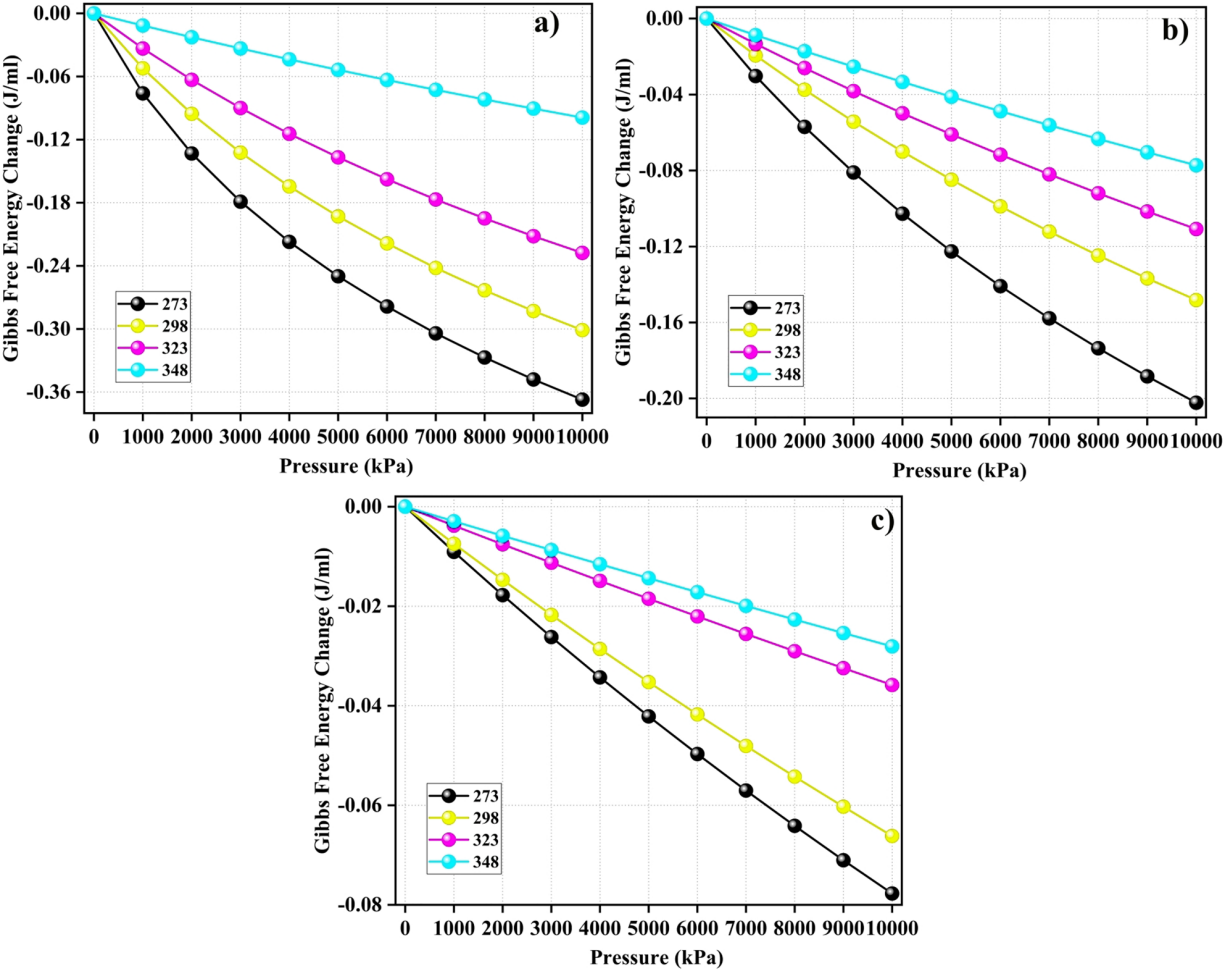
$\Delta G$ values associated with (**a**) CO$_2$, (**b**) CH$_4$, and (**c**) N$_2$ adsorption on the surface of model coal-derived asphaltene at various temperatures.

**Figure 10:**
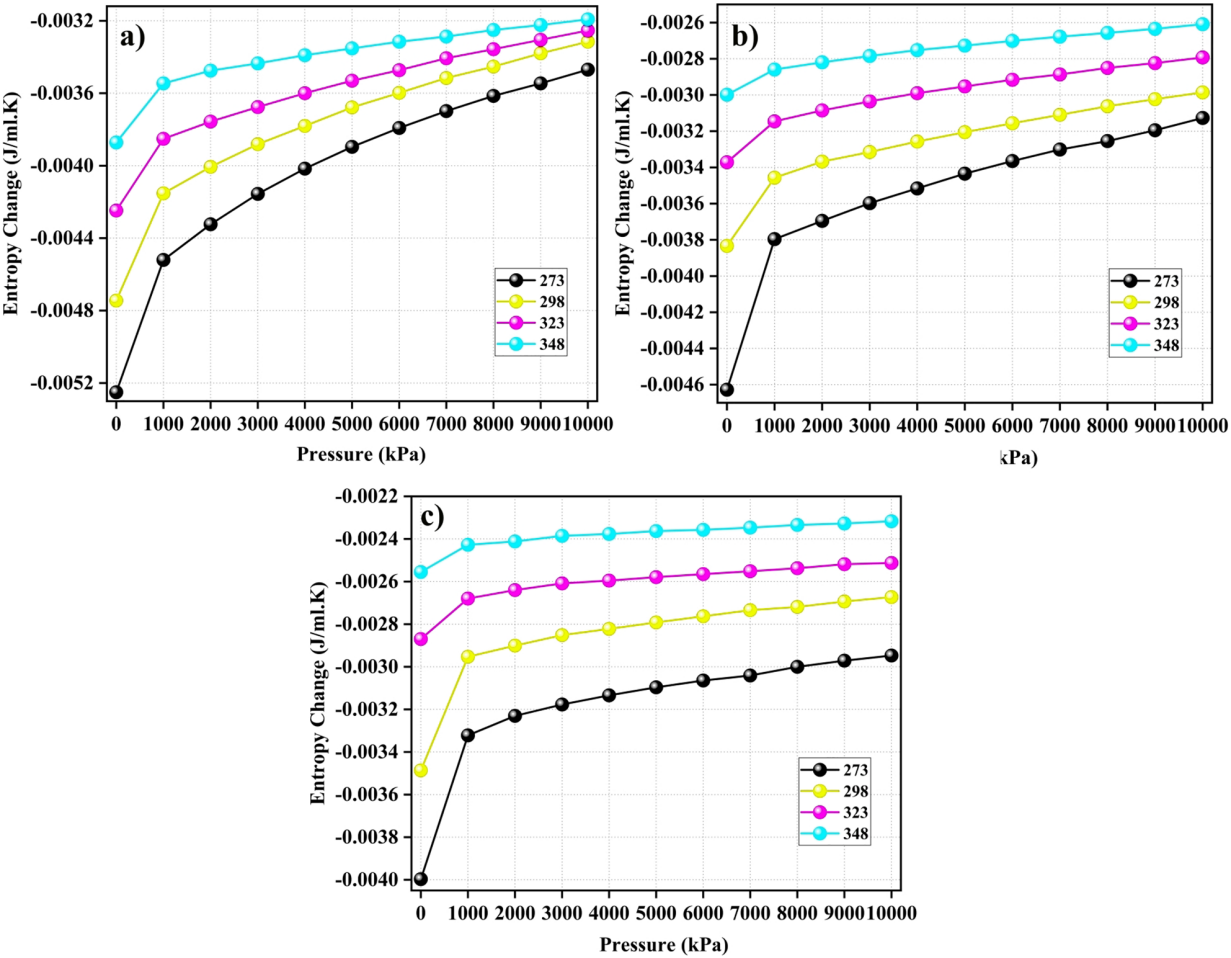
Entropy changes during the adsorption of (**a**) CO$_2$, (**b**) CH$_4$, and (**c**) N$_2$ on model coal-derived at various temperatures.

As demonstrated in Fig. 9, an examination of the changes in Gibbs free energy during the adsorption process of CO$_2$, CH$_4$, and N$_2$ on the adsorbents indicates the thermodynamic spontaneity of these processes, as evidenced by their negative values. Despite the unfavorable effect of increasing the temperature on the adsorption process, the influence of pressure is favorable for enhancing the spontaneity of adsorption. This is due to the higher likelihood of gas molecules adhering to the surface of the adsorbent under high pressures^11^. Notably, for CO$_2$ gas, $\Delta G$ has significantly higher absolute values than those of other gases, highlighting a greater level of spontaneity in CO$_2$ compared to CH$_4$ and N$_2$.

**Entropy**

The entropy change ($\Delta S$) is a valuable tool for understanding adsorption behavior, as it indicates the degree of system disorder^11^. Studying the changes in entropy concerning adsorption capacity provides valuable insights into the arrangement of molecules on the surface of the adsorbent. This analysis provides crucial information regarding the adsorption process^46^. The entropy change $\Delta S$ (J.mL$^{-1}$K$^{-1}$) is defined using the following formula^11^:

10 $$\begin{aligned} \Delta S = \frac{\Delta H - \Delta G}{T} \end{aligned}$$

T represents the absolute temperature and $\Delta H = -Q_{st}$^43^. Changes in entropy result from the interaction of different molecular movements, such as rotational degrees of freedom, vibrational modes, and the translational motion of gas molecules^46^. The $\Delta S$ values associated with gas adsorption on PA3 are depicted in Fig. 10. In general, the absolute magnitude of $\Delta S$ decreases with increasing pressure. At lower pressures, the gas molecules showed enhanced interactions with the surfaces of the adsorbents. This behavior arises because gas molecules, once adsorbed, can absorb additional energy from the environment and then detach from the adsorbent surface. If a gas exhibits a $\Delta S$ value below zero, it indicates a shift in the adsorption process. This shift progressed from disorder to order, resulting in an organized configuration of gas molecules on the surface of the adsorbent^11^. Remarkably, the most significant absolute change in entropy occurred during the adsorption of all gases at the lowest temperature (273 K) with increasing pressure. This suggests that the impact of the entropy change diminishes as the temperature increases.

### Density functional theory

#### Theoretical reactivity parameters and molecular electrostatic potential

The difference in the electron energy levels between the highest occupied molecular orbital (HOMO) and lowest unoccupied molecular orbital (LUMO) can be considered a reliable indicator of changes in electronic properties and chemical reactivity. The reactivity parameters studied were the chemical potential $\mu$ (eV), electrophilicity $\omega$ (eV), and chemical hardness $\eta$ (eV). Hardness is a parameter defined by the charge and polarizability of the system. It causes changes in the energy of the HOMO and LUMO, consequently altering the band gap. Chemical hardness is defined as an indicator of a molecule’s resistance to charge transfer to the environment. This implies that the chemical hardness of a molecule has an inverse relationship with the charge transfer. The higher the chemical hardness of a molecule, the lower the chance of charge transfer and interaction of the molecule with its environment. The chemical potential is an indicator of the stability of the system and shows the tendency of the system to remain in its current conditions^50^. Koopman’s theory calculates the chemical potential and hardness using the following equations^31^:

11 $$\begin{aligned} \eta&= \frac{(IP - EA)}{2} = - \frac{1}{2} \Bigl (E_{HOMO} - E_{LUMO}\Bigr ) \end{aligned}$$

12 $$\begin{aligned} \mu&= \frac{(IP+EA)}{2} = \frac{1}{2} \Bigl (E_{HOMO} + E_{LUMO}\Bigr ) \end{aligned}$$

IP (eV) refers to the ionization energy, which is equivalent to E$_{HOMO}$ (eV), and EA (eV) represents the electron affinity. EA is proportional to -E$_{LUMO}$ (eV). Parr and his colleagues proposed a parameter called electrophilicity $\omega$ (eV), which determines whether a molecule is an electron acceptor or donor. Larger values of the electrophilicity parameter of the molecule indicate that the molecule is an electron acceptor. Electrophilicity can be defined as follows^51^:

13 $$\begin{aligned} \omega = \frac{\mu ^2}{2\eta } \end{aligned}$$

The values of the HOMO and LUMO orbital energies, band gaps, hardness, chemical potentials, and electrophilicity of functionalized PA3 calculated by the DFT B3LYP/6-311++G(d,p) level of theory are shown in Table 2. The chemical hardness values for the asphaltene structures follow the order:

PA3S > PA3O > PA3N > PA3C,

while the chemical potential values are as follows:

PA3N > PA3C > PA3O > PA3S.

These results indicate that the PA3S structure with the highest hardness also has the lowest chemical potential value. This suggests that the structure is stable and less likely to transfer charge or participate in reactions. The structures of PA3C and PA3N have the lowest hardness and the highest chemical potential, indicating their tendency to transfer charge. These findings are consistent with the adsorption energy data. The electrophilicity values follow the order:

PA3S > PA3C > PA3O > PA3N.

**Table 2:** The HOMO and LUMO orbital energies, band gap, hardness, chemical potential, and electrophilicity of functionalized PA3 were calculated using DFT at the B3LYP/6-311++G(d,p) level of theory.

Reactivity Parameters	PA3C	PA3S	PA3O	PA3N
$E_{HOMO}$ (eV)	− 4.999	− 5.186	− 5.113	− 4.945
$E_{LUMO}$ (eV)	− 1.813	− 1.853	− 1.789	− 1.672
$\Delta E_{HOMO-LUMO}$ (eV)	3.186	3.333	3.324	3.273
$\eta$ (eV)	1.593	1.666	1.662	1.636
$\mu$ (eV)	− .401	− 3.521	− 3.451	− 3.308
$\omega$ (eV)	3.630	3.720	3.582	3.344
Dipole moment (Debye)	0.657	1.631	1.713	1.412

**Figure 11:**
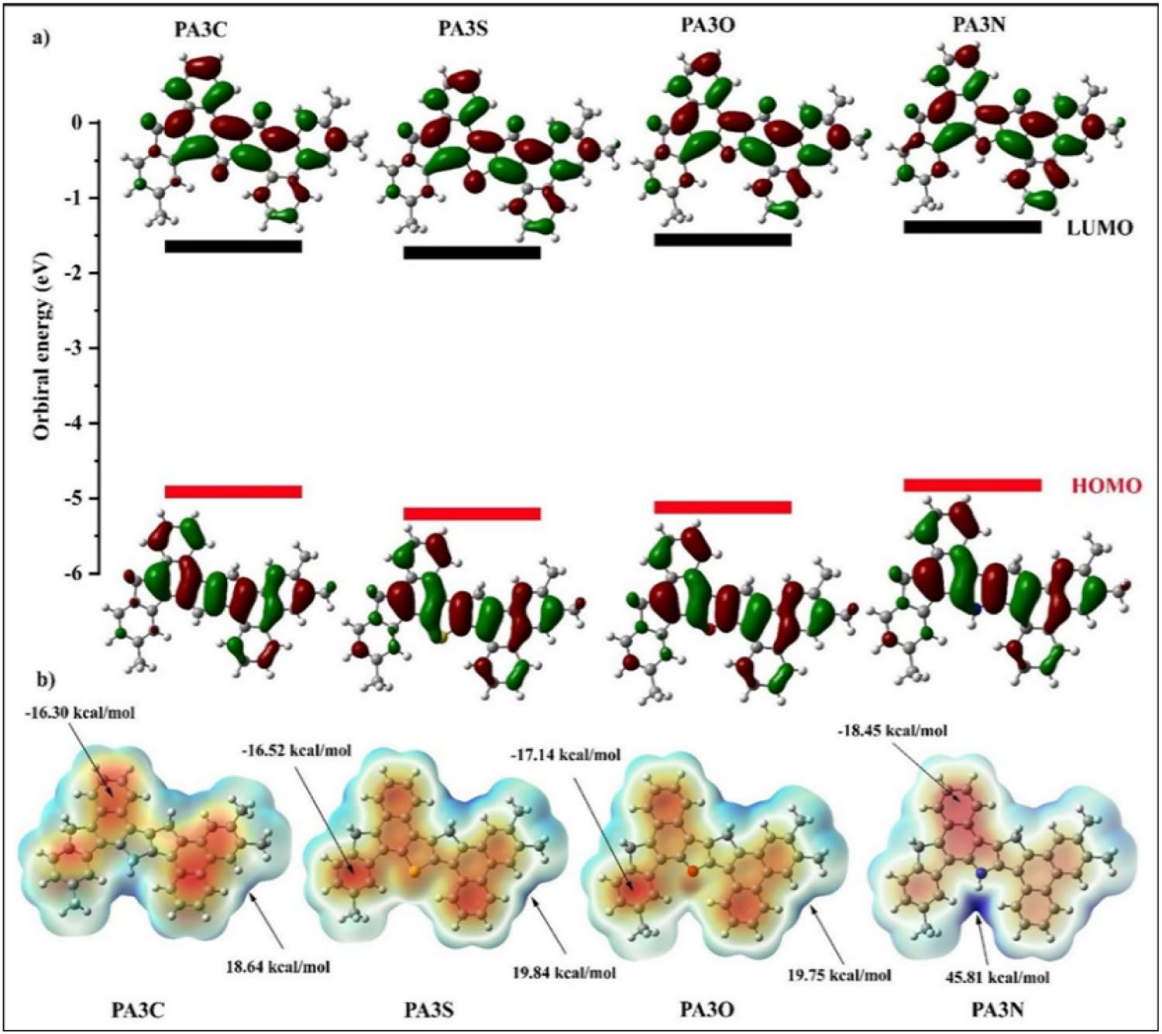
The diagram of HOMOs and LUMOs of functionalized PA3 using the B3LYP functional and the 6-311++G(d,p) basis set. Additionally, it includes molecular electrostatic potential (MEP) maps for the functionalized asphaltene (PA3) molecule, indicating regions of maximum (red) and minimum (blue) electron density.

Molecular electrostatic potential analysis provides information about the active sites involved in the interaction between the adsorbent and the adsorbate. Color schemes often highlight these possibilities. In this map, as depicted in Fig. 11, the red color indicates high electron density values (nucleophilic sites), while the blue color indicates low electron density values (electrophilic sites). Therefore, by using ESP, it is possible to understand which part of a molecule can participate more in interactions and, to some extent, help understand the reaction mechanism^52^. The 45.81 kcal/mol region on the PA3N molecule enhances its potential to interact with CO$_2$, N$_2$, and CH$_4$ compared to PA3C, PA3O, and PA3S.

When the methine group was used instead of a heteroatom in asphaltene, the electrostatic potential map showed that the methine group was electrophilic. This was reflected in the highly negative adsorption energies observed for the adsorption of gas molecules on their structure. Sulfur and oxygen atoms are nucleophilic as heteroatoms in the asphaltene structure; ESP plots for CO$_2$, CH$_4$, and N$_2$ are shown in Fig. S4. CO$_2$ adsorption on asphaltene structures has the highest (absolute) adsorption energy, which is attributed to the interaction between the highly electrophilic oxygen in CO$_2$ and the nucleophilic sites in the adsorbents. The structures of PA3N and PA3C have the lowest band gaps, indicating that these two structures are more reactive. This finding is consistent with the adsorption energy and chemical potential data.

Electron donors and acceptors were studied using electron density difference analysis. Here, we investigate the electron density differences to gain a better understanding of the interaction between the adsorbate and the surface. This difference is defined as follows^53^:

14 $$\begin{aligned} \Delta \rho (r) = \rho _{\text {Surf-Adsor}}(r) - \rho _{\text {Surf}}(r) - \rho _{\text {Adsor}}(r) \end{aligned}$$

$\rho _{\text {Surf-Adsor}}(r)$ denotes the electronic density of the surface covered by the adsorbate, while $\rho _{\text {Surf}}(r)$ and $\rho _{\text {Adsor}}(r)$ denote the densities of the surface and the adsorbate, respectively. This difference demonstrates the redistribution of the electronic density that occurs as a result of adsorption. The plots in Fig. S5 show that the adsorbates have a positive electron density difference, indicating that they are acceptors, while the asphaltenes are electron donors. The functional groups on the asphaltene structures (N, O, S) also have a positive electron density difference, indicating that they are acceptors. In contrast, the C group at the same position exhibits a negative electron density difference.

#### Adsorption energy calculation

**Figure 12:**
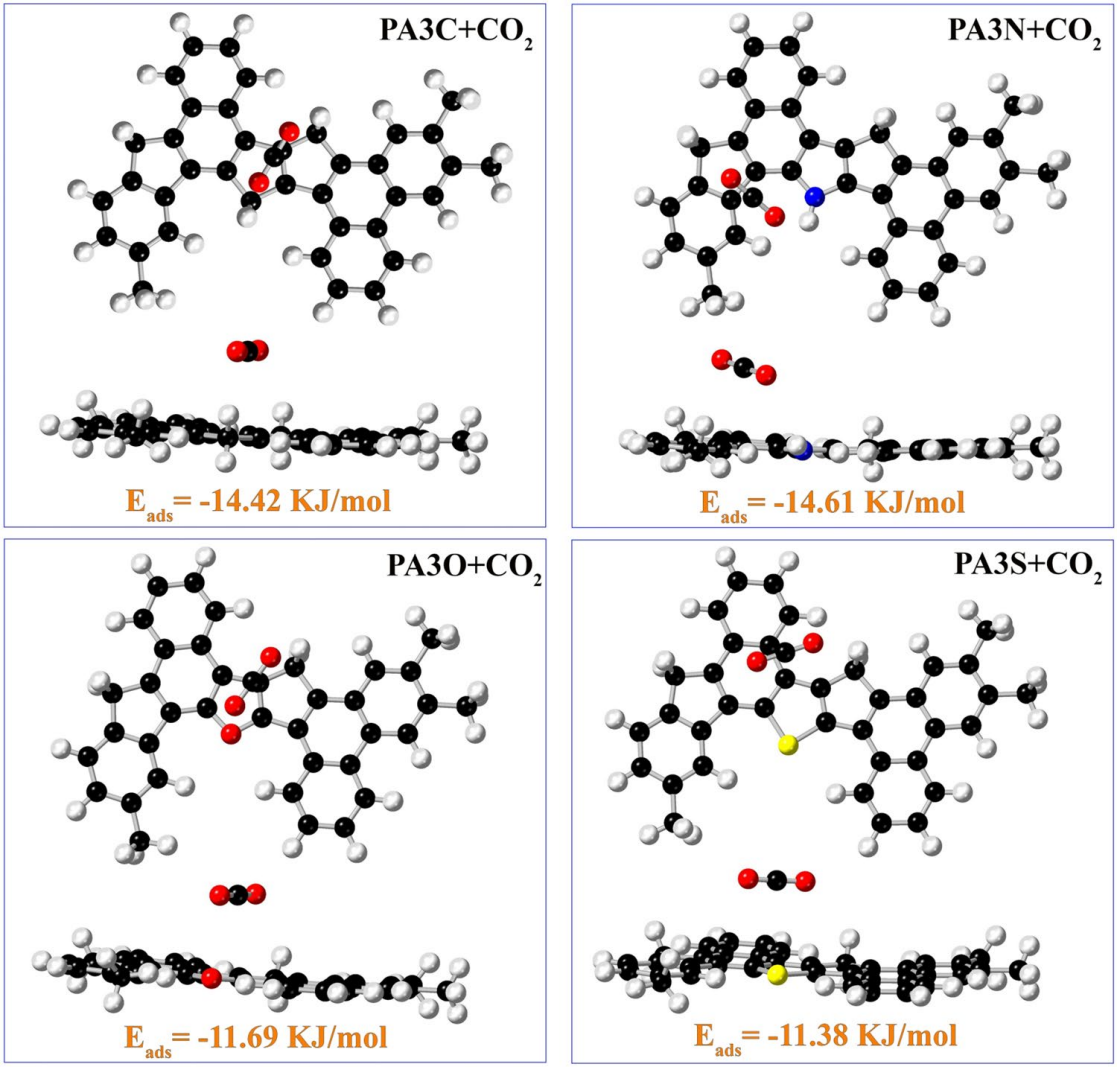
Stable adsorption configurations of the CO$_2$ molecule adsorbed on the surface of asphaltene fragments.

The adsorption energy $E_{ads}$ (KJ/mol) was calculated to further evaluate the adsorption mechanism of CO$_2$, CH$_4$, and N$_2$ gas molecules on asphaltene molecules derived from the model coal.

15 $$\begin{aligned} E_{ads} = E_{a+s} - E_s - E_a, \end{aligned}$$

where $E_a$ represents the energy of the adsorbates, $E_s$ is the energy of functionalized PA3, and $E_{ads}$ represents the energy of the adsorbate-functionalized PA3 structure complex. Figure 12 shows the optimized structures of molecule adsorption on carbon oxide on the four asphaltene structures, along with their adsorption energies. Other structures are shown in Figs. S6, S7. Additionally, Table S5, displaying all adsorption energies, is included. For the adsorption of CO$_2$ and N$_2$ molecules, the adsorption energy values follow the order:

PA3N < PA3C < PA3O < PA3S.

For the adsorption of CH$_4$ on coal-derived asphaltenes, the adsorption energy values follow the order:

PA3N < PA3O < PA3S < PA3C.

**Figure 13:**
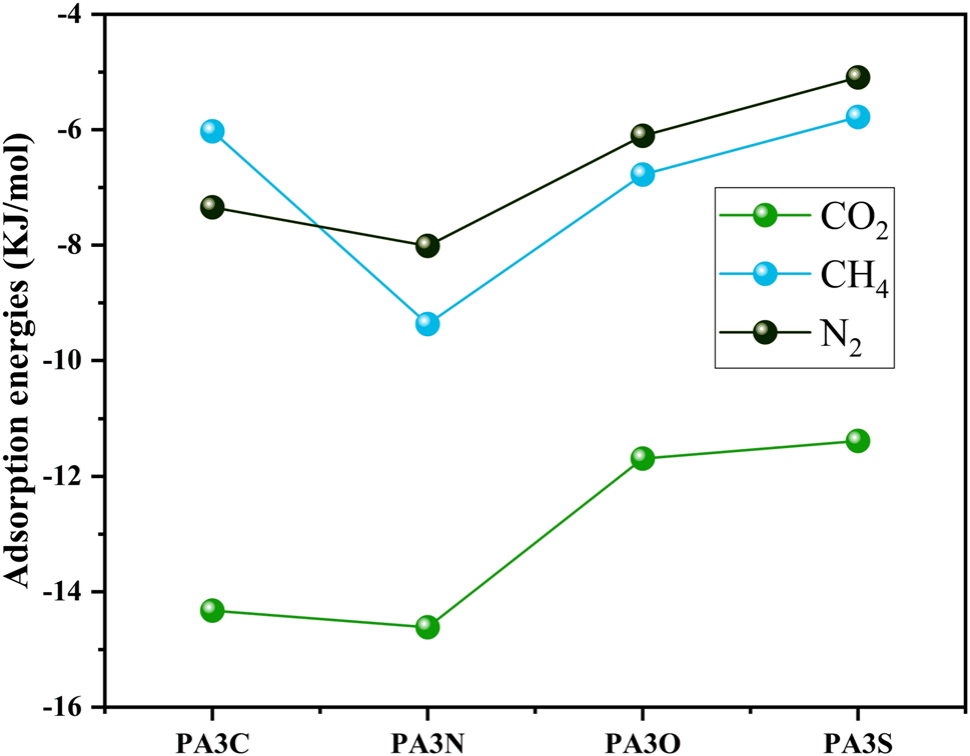
Examination of adsorption energies ($E_{ads}$ ) of CO$_2$ ,CH$_4$, and N$_2$ by the surface of asphaltene fragments.

The adsorption energy values indicate the physical adsorption of gas molecules onto all the asphaltene structures investigated in this study. Figure 13 illustrates an analysis of the adsorption energies ($E_{ads}$) of CO$_2$, CH$_4$, and N$_2$ on the PA3 surface. The highest (absolute) adsorption energy belongs to the adsorption of CO$_2$ on PA3N due to the $\pi -\pi$ interaction between CO$_2$ and the aromatic rings of asphaltene, as well as van der Waals interactions between the adsorbate and adsorbent. The adsorption energy for the adsorbates follows the order N$_2$ > CH$_4$ > CO$_2$. Furthermore, other adsorbent-adsorbate structures also exhibit $\pi -\pi$ interactions, while hydrogen bonding is less commonly observed. The adsorption energies were calculated at the $\omega$B97XD/6-31+G(d,p) level of theory.

#### Nature of interactions

Non-covalent interactions are utilized to comprehend the nature of intermolecular forces between molecules. The NCI relies on the electron density and reduced density gradient (s), as shown in the following equation^54,55^:

16 $$\begin{aligned} s = \frac{1}{2(3\pi ^2)^{1/3}}\frac{\nabla \rho }{\rho ^{\frac{4}{3}}} \end{aligned}$$

**Figure 14:**
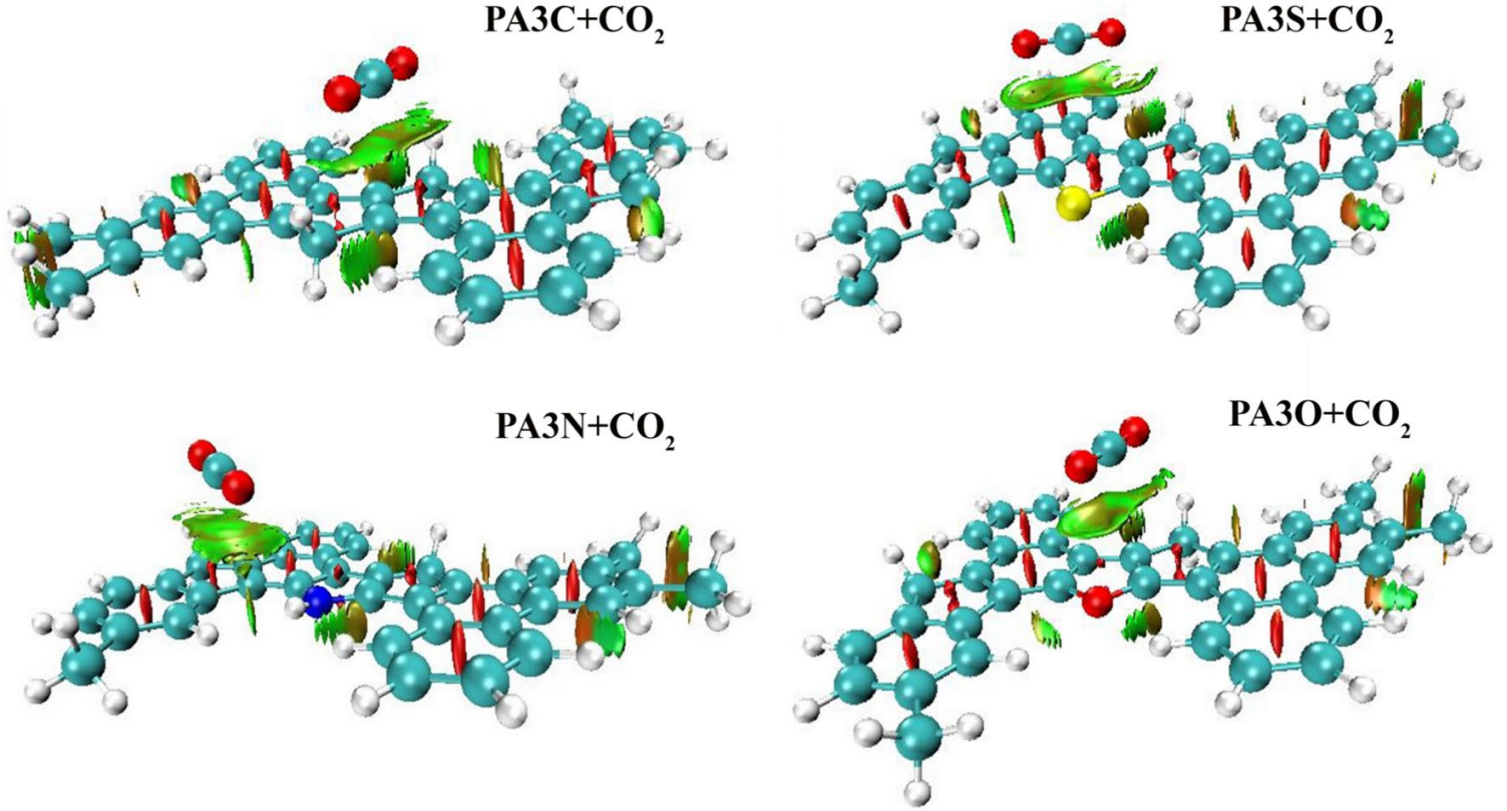
Three-dimensional representation of the analysis of non-covalent interactions (NCI) for CO$_2$ adsorption on PA3C, PA3S, PA3N, and PA3O at the 6-31+G(d,p) level of theory, illustrating van der Waals interactions. Diagrams were created using isosurfaces with RDG 0.6 au. The colors are marked from -0.05 < (sign $\lambda _2$)$\rho$ < 0.05 au.

This analysis can be used to determine intermolecular forces, such as hydrogen bonding, van der Waals interactions, and repulsive steric interactions. Two general methods were used for this analysis. In the first method, a graph is generated with plots of the reduced density gradient (s) versus (sign $\lambda _2$)$\rho$, where (sign $\lambda _2$)$\rho$ represents the electron density multiplied by the sign of the second Hessian eigenvalue ($\lambda _2$). The value of (sign $\lambda _2$)$\rho$ is useful for predicting the nature of the interaction. RDG and sign ($\lambda _2$) (r)$\rho$(r) define specific areas. Based on ($\lambda _2$) and ($\rho$), sign($\lambda _2$)$\rho$ < 0 indicates hydrogen bonding here, sign($\lambda _2$)$\rho$ > 0 indicates a repulsive interaction, and sign($\lambda _2$)$\rho$ $\approx$ 0 implies van der Waals interactions^54^. In the second method, intermolecular interactions in the NCI method can be visualized by using the gradient isosurface in the real space of the molecules. In the 3D visualization of NCI isosurfaces, green represents van der Waals interactions, blue indicates hydrogen bonding, and red signifies repulsive steric interactions^55,56^. To investigate intermolecular interactions, we utilized two- and three-dimensional non-covalent interaction analyses. The analysis of non-covalent interactions in three dimensions shows the van der Waals interactions (green color) for the adsorption of gas molecules on asphaltene structures. Figure 14 illustrates the reduced density gradient isosurfaces representing non-covalent interaction (NCI) regions during the adsorption of CO$_2$ on PA3C, PA3S, PA3N, and PA3O at the $\omega$B97XD/6-31+G(d,p) level of theory. The isosurfaces were constructed with an RDG value of 0.6 au. With a color scaling range of -0.05 < (sign $\lambda _2$)$\rho$ < 0.05 au. Other non-covalent interaction structures are depicted in the Supplementary Information. NCI analysis of the adsorption of CH$_4$ and N$_2$ on coal-derived asphaltenes is depicted in Figs. S8, S9. NCI analysis in two dimensions for the adsorption of CH$_4$, CO$_2$, and N$_2$ on the PA3C, PA3S, PA3N, and PA3O surfaces indicates van der Waals interactions (Fig. S10). According to the adsorption energies and optimized structures obtained from DFT calculations, we observed that the adsorption is physical in nature, indicating the absence of covalent interactions (chemical adsorption) between the adsorbates and adsorbents. Overall, the analysis of non-covalent interactions suggests physical interactions such as hydrogen bonding, van der Waals forces, and steric repulsion.

### Molecular dynamics

#### Displacement analysis

**Figure 15:**
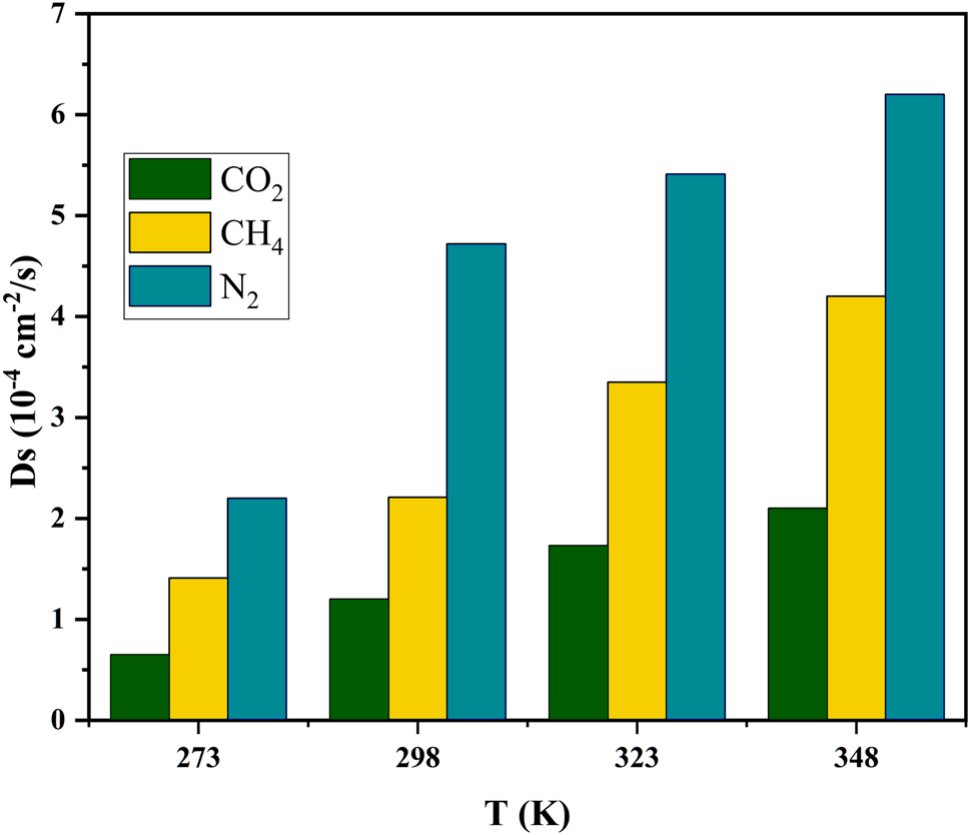
Self-diffusion coefficients of CO$_2$, CH$_4$, and N$_2$ for adsorption onto the model coal-derived asphaltene as a function of temperature T = 273 K, 298 K, 323 K, and 348 K.

Assessing the adsorption process requires a comprehensive understanding of the dynamic interactions between a gas and asolid sorbent. Self-diffusion coefficients provide valuable insights to assist in this process^14^. We used Einstein’s method to calculate the self-diffusion coefficients $D_S$ (cm$^{-2}$.s$^{-1}$) of gases within the nanopores of PA3. This method is expressed as follows^25^:

17 $$\begin{aligned} D_S = \frac{1}{6} \frac{d}{dt} \sum _{i=1}^{n} \langle |{\textbf{r}}_i(t) - {\textbf{r}}_i(0)|^2 \rangle \end{aligned}$$

here, $|{\textbf{r}}_i(t) - {\textbf{r}}_i(0)|^2$ represents the mean square displacement (MSD) of particles over time, and the angular brackets denote the ensemble average. A factor of 1/6 arises from the three-dimensional space, where each dimension contributes to the overall diffusion^8^. The self-diffusion coefficients at temperatures of 273 K, 298 K, 323 K, and 348 K for CO$_2$, CH$_4$, and N$_2$ within the model asphaltene are graphically illustrated in Fig. 15. It is evident that CO$_2$ has a significantly lower self-diffusion coefficient compared to the other two gases. The lower value of Ds for CO$_2$ confirms its stronger adsorption onto the model asphaltenes, indicating that CO$_2$ molecules exhibit more potent adsorption interactions than other gases.

#### Radial distribution function

Radial Distribution Function (RDF), a widely employed and effective technique, serves as a valuable tool for exploring interactions within molecular systems^57^. In particular, this method explores the potential existence of a specific chemical species ($\alpha$) that may be present near another chemical group ($\beta$) separated by a defined distance (r). A representation of the RDF is outlined below^31^:

18 $$\begin{aligned} g_{\alpha \beta }(r) = \frac{V}{N_{\alpha }N_{\beta }} \Bigl (\sum _{i=1}^{N_\alpha } \frac{n_i \beta (r)}{4\pi r^2 \Delta r}\Bigr ) \end{aligned}$$

The provided formula includes various variables: V (Å$^3$) represents the volume of the system, $N_\alpha$ and $N_\beta$ denote the numbers of particles $\alpha$ and $\beta$ respectively, and the notation $n_i\beta (r)$ signifies the aggregated count of particles belonging to type $\beta$ within a spherical shell located at a distance r (Å) from a particle of type $\alpha$. Within the context of adsorption, RDF represents the ratio of density in a confined region to the average density of the entire system. This provides insight into the structure of the adsorption sites, especially for CO$_2$ adsorption on asphaltene molecules^1,8^.

**Figure 16:**
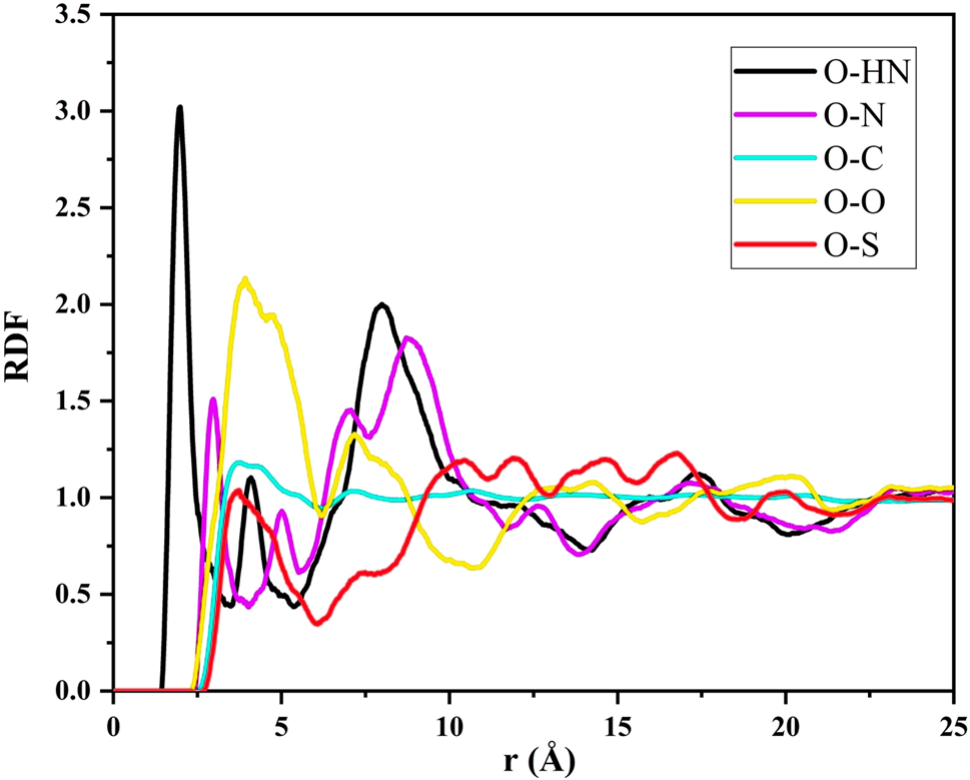
RDF between CO$_2$ and the PA3 surface with the following interactions: O-HN (O from CO$_2$ and H from PA3N), O-N (O from CO$_2$ and N from PA3N), O-C (O from CO$_2$ and C from all PA3 molecules), O-O (O from CO$_2$ and O from PA3O), O-S (O from CO$_2$ and S from PA3S).

Figure 16 illustrates the RDF plots of the interactions between CO$_2$ and the PA3 surface. These interactions represent the following pairs: O-HN (O from CO$_2$ and H from PA3N), O-N (O from CO$_2$ and N from PA3N), O-C (O from CO$_2$ and C from all PA3 molecules), O-O (O from CO$_2$ and O from PA3O), and O-S (O from CO$_2$ and S from PA3S). The RDF analysis revealed significant interactions, notably between the oxygens of CO$_2$ molecules and hydrogen atoms attached to the nitrogen in PA3N, as evidenced by a prominent peak at 2 Å. Additionally, noteworthy peaks were observed for the nitrogen in PA3-N, oxygen in PA3-O, and sulfur in PA3-S. These findings collectively emphasize the significant contributions of CO$_2$ oxygen atoms to the adsorption process of the model asphaltenes.

## Conclusion

In this study, we employed grand canonical Monte Carlo, molecular dynamics, and density functional theory to analyze the adsorption behavior of CH$_4$, CO$_2$, and N$_2$ gases on a model asphaltene. We aimed to understand the mechanisms underlying competitive adsorption at various temperatures and pressures. We made several specific findings. It was found that an increase in temperature resulted in reduced adsorption, whereas an increase in pressure had a positive effect. Competitive adsorption favors CO$_2$ over CH$_4$ and N$_2$, especially at elevated temperatures. The adsorption of all gases exhibited isosteric heat values below 10 kcal/mol, indicating a physisorption process. CO$_2$ exhibits an advantage over the other two gases, as confirmed by the higher absolute values of the surface potential. All the adsorption processes were thermodynamically spontaneous, with CO$_2$ having the highest (absolute) Gibbs free energy. Entropy changes reflect the transition to an ordered phase during the adsorption process. The self-diffusion coefficients were the lowest for CO$_2$, indicating stronger interactions with the adsorbate. The DFT results indicate that the adsorption of CO$_2$ on asphaltenes derived from coal exhibited the highest (absolute) adsorption energies. Additionally, the adsorbent PA3N was found to be the most suitable for CO$_2$-ECBM and N$_2$-ECBM. Analysis of non-covalent interactions revealed that the primary interactions between the coal-derived asphaltenes and the CO$_2$, N$_2$, and CH$_4$ adsorbates are van der Waals forces. These findings highlight the complexity and significance of adsorption processes. Our findings provide a comprehensive understanding of the adsorption behavior of these gases, which could serve as a foundation for future research in this field. The insights gained from this study could be particularly useful for researchers investigating the adsorption properties of other gases and materials.

## Supplementary Information

Supplementary Information.

## Data Availability

The data will be available from the corresponding author upon a reasonable request.
