# Supplementary material for: Competitive adsorption of CO2, N2, and CH4 in coal-derived asphaltenes, a computational study
Source: Sci Rep. 2024 Apr 1;14:7664. doi: 10.1038/s41598-024-58347-6 (PMC11379724; doi:10.1038/s41598-024-58347-6)
Supplement: Supplementary file 1 — Supplementary Information. [file 41598_2024_58347_MOESM1_ESM.docx]

Competitive Adsorption of CO2, N2, and CH4 in Coal-Derived Asphaltenes, A Computational Study

Farshad Mirzaee Valadi^1,^ ^+^, Mohammad Pasandideh-Nadamani ^2,^ ^+^, Mozafar Rezaee^3,^ ^++^, Abdolhalim Torrik^4, ++^, Mohammad Mirzaie^1^,Ayoob Torkian^1,*^

^1^Water and Energy Research Center, Sharif University of Technology, Tehran, Iran

^2^Department of Chemistry, University of Mazanderan, Babolsar, Mazanderan, Iran

^3^Department of Chemistry, Iran University of Science and Technology, Tehran, Iran

^4^Department of Physical and Computational Chemistry, Shahid Beheshti University, Tehran, Iran

Author Contributions:

+these authors contributed equally to this work
++these authors contributed equally to this work

^*^ Corresponding Author’s E-mail Address: torkian@sharif.edu

**Acronyms**

CBM Coalbed methane recovery

ECBM Enhanced coalbed methane recovery

RDF Radial distribution function

NCI Non Covalent Interaction

MD Molecular dynamics

DFT Density functional theory

HOMO Highest occupied molecular orbital

LUMO Lowest unoccupied molecular orbital

COMPASS Condensed-phase optimized molecular potentials for atomistic simulations studies

ESP Electrostatic potential

NVT Constant pressure and volume, Constant temperature and volume

PA3C Model coal-derived asphaltene molecule with C atom

PA3N Model coal-derived asphaltene molecule with N atom

PA3O Model coal-derived asphaltene molecule with O atom

PA3S Model coal-derived asphaltene molecule with S atom

Variables / Letters

P Pressure

D_s_ Self-diffusion coefficient

q Adsorption Charging

K_H_ Henry’s constant

T Temperature

R_g_ Universal gas constant

Q_st_ Isosteric heat

S Selectivity parameter

Greek letters

μ Electronic Chemical Potential

ω Electrophilicity Power

η Chemical Hardness

Ω Surface potential

Subscripts

α α moieties

β β moieties


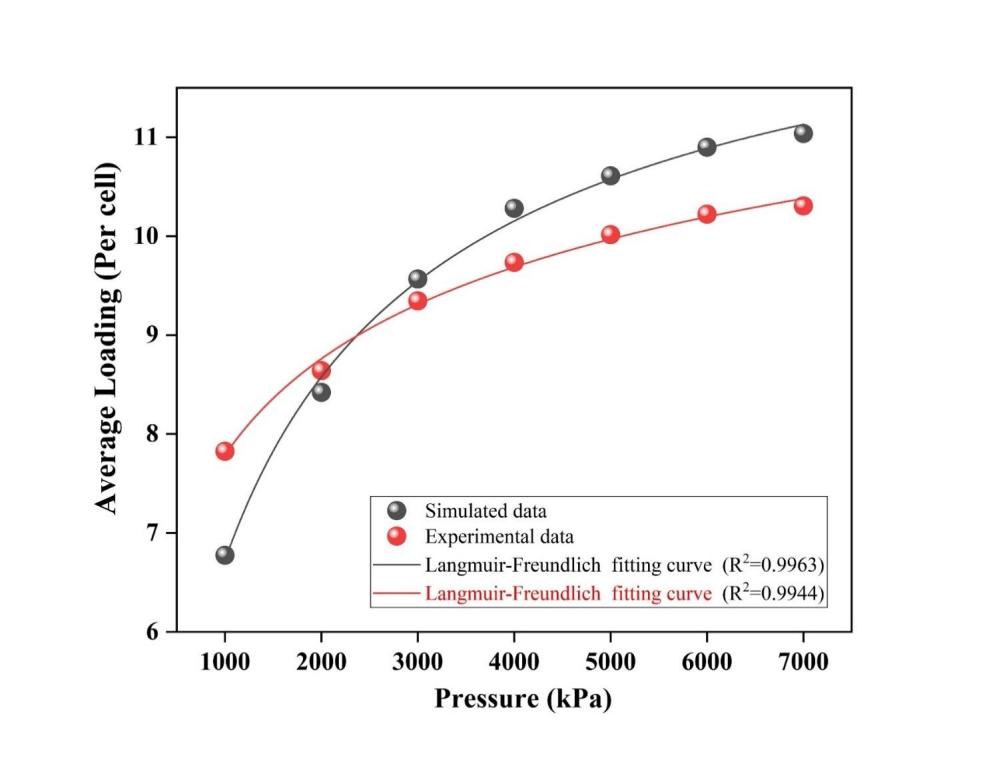


**Figure S1**. Comparisons between the simulated and experimental adsorption isotherms for CO_2_ at 298 K.

**COMPASS Force field**

The COMPASS force field is an all-atom force field that is built on ab initio and experimental data optimization. The following equation is a description of its potential function[2]:

$$E_{pot}=\sum_{b} \left[ K_{2}\left( b-b_{0} \right)^{2}+K_{3}\left( b-b_{0} \right)^{3}+K_{4}\left( b-b_{0} \right)^{4} \right]+\sum_{\theta} H_{2}\left( \theta-\theta_{0} \right)^{2}+H_{3}\left( \theta-\theta_{0} \right)^{3}+H_{4}\left( \theta-\theta_{0} \right)^{4}+\sum_{\phi} \left[ V_{1}\left[ 1-coscos\left( \phi-\phi_{1}^{0} \right) \right]+V_{2}\left[ 1-coscos\left( 2\phi-\phi_{2}^{0} \right) \right]+V_{3}\left[ 1-coscos\left( 3\phi-\phi_{3}^{0} \right) \right] \right]+\sum_{\chi} K_{\chi}\chi^{2}+\sum_{b} \sum_{b'} F_{bb'}\left( b-b_{0} \right)\left( b^{'}-{b'}_{0} \right)+\sum_{\theta} \sum_{\theta'} F_{\theta\theta'}\left( \theta-\theta_{0} \right)\left( \theta^{'}-{\theta'}_{0} \right)+\sum_{b} \sum_{\theta} F_{b\theta}\left( b-b_{0} \right)\left( \theta-\theta_{0} \right)+\sum_{b} \sum_{\phi} \left( b-b_{0} \right)\left[ V_{1}cos\phi+V_{2}cos2\phi+V_{3}cos3\phi\right]+\sum_{b'} \sum_{\phi} \left( b^{'}-{b^{'}}_{0} \right)\left[ V_{1}cos\phi+V_{2}cos2\phi+V_{3}cos3\phi\right]+\sum_{\theta} \sum_{\phi} \left( \theta-\theta_{0} \right)\left[ V_{1}cos\phi+V_{2}cos2\phi+V_{3}cos3\phi\right]+\sum_{\phi} \sum_{\theta} \sum_{\theta'} F_{\phi\theta\theta'}cos\phi\left( \theta-\theta_{0} \right)\left( \theta^{'}-{\theta'}_{0} \right)+\sum_{i>j} \frac{q_{i}q_{j}}{{\varepsilon r}_{ij}}+\sum_{i>j} \left[ \frac{A_{ij}}{r_{ij}^{9}}+\frac{B_{ij}}{r_{ij}^{6}} \right]$$

The symbols with the subscript 0 represent all equilibrium parameters that are constant. Variables with no subscript are variables that are dependent on atom locations. The nomenclature of the COMPASS force field symbols is listed in **Table S1.**

**Table S1**. Nomenclature of the COMPASS force field symbols.

| **Symbol** | **Definition** |
| --- | --- |
| $K_{2},K_{3},K_{4}$ | bond stretch elastic constants |
| $b$ | Chemical bond length |
| $\theta$ | Bond angle |
| $H_{2},H_{3},H_{4}$ | Angle elastic constants |
| $V_{1},V_{2},V_{3}$ | Torsion angle elastic constants |
| $\phi$ | The dihedral angle formed by four adjacently linked atoms that are not in the same plane. |
| $K_{\chi}$ | Out-of-plane bending elastic constant |
| $\chi$ | Out-of-plane bending angle |
| $F_{bb'}$ | Stretch–stretch coupling elastic constant |
| $F_{\theta\theta'}$ | Bending–bending coupling elastic constant |
| $F_{b\theta}$ | Stretch–bending coupling elastic constant |
| $F_{\phi\theta\theta'}$ | Torsion–bending–bending coupling elastic constant |
| $\varepsilon$ | Effective dielectric constant |
| $q_{i}q_{j}$ | Atom charges |
| $r_{ij}$ | Distance between two atoms |
| $A_{ij},B_{ij}$ | Lennard-Jones potential parameters |


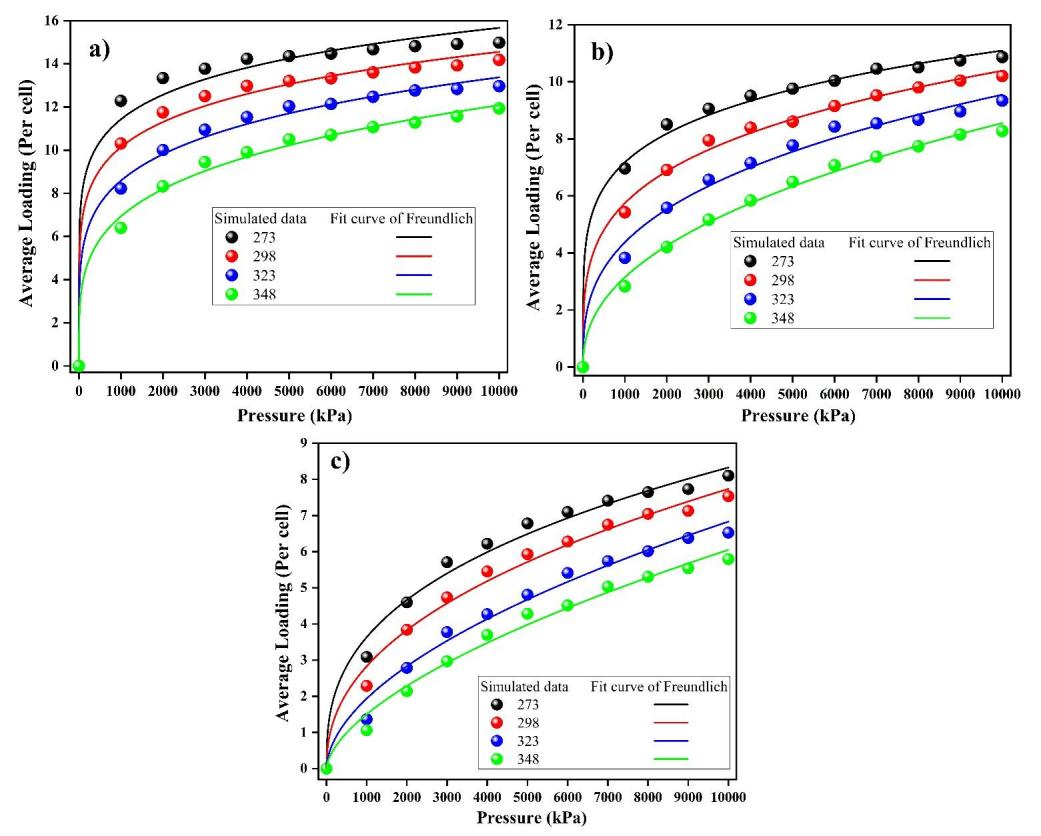


**Figure S2.** Freundlich Isotherms for a) CO_2_, b) CH_4_, and c) N_2_ on model coal-derived asphaltene at different temperatures.


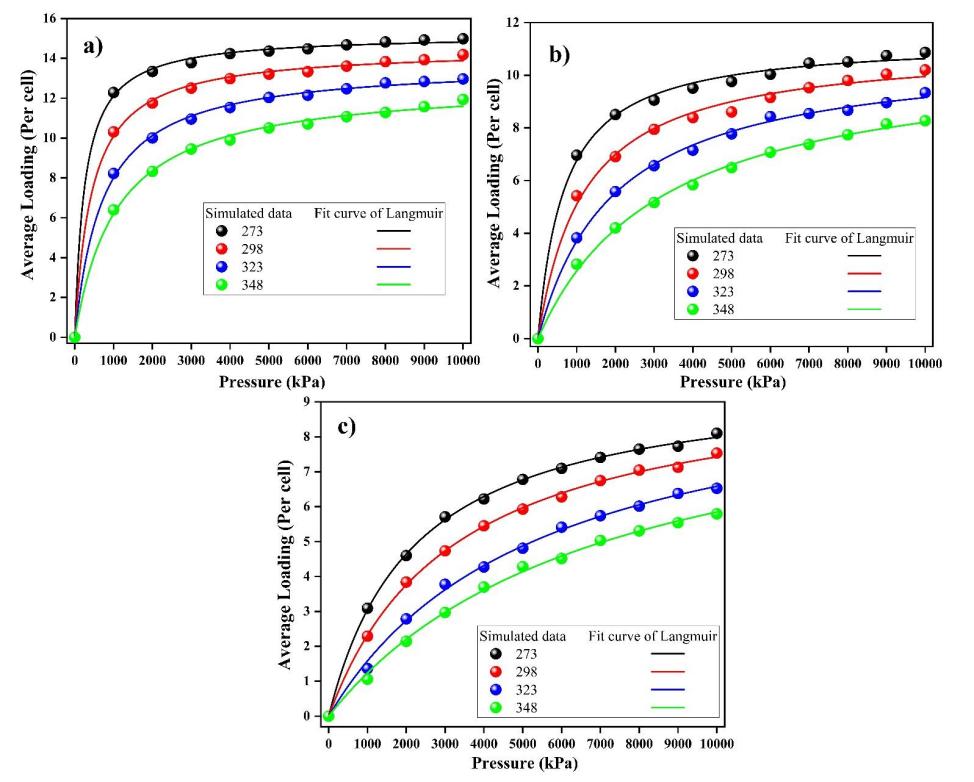


**Figure S3.** Langmuir Isotherms for a) CO_2_, b) CH_4_, and c) N_2_ on model coal-derived asphaltene at different temperatures.

**Table S2.** Adsorption Isotherm Coefficients for CO_2_

| **CO2** | **Langmuir model** | | | **Freundlich model** | | | **Langmuir-Freundlich model** | | | |
| --- | --- | --- | --- | --- | --- | --- | --- | --- | --- | --- |
| T (K) | a | b (kPa^-1^) | R^2^ | k | n | R^2^ | a | b (kPa^-1^) | n | R^2^ |
| 273 | 15.19 | 0.0039 | 0.9990 | 4.43 | 7.29 | 0.9699 | 16.53 | 0.0791 | 1.91 | 0.9998 |
| 298 | 14.49 | 0.0023 | 0.9984 | 3.42 | 6.36 | 0.9860 | 16.09 | 0.0302 | 1.70 | 0.9997 |
| 323 | 13.76 | 0.0013 | 0.9988 | 2.27 | 5.19 | 0.9923 | 14.93 | 0.0075 | 1.35 | 0.9998 |
| 348 | 12.83 | 0.0004 | 0.9978 | 1.30 | 4.14 | 0.9914 | 14.43 | 0.0056 | 1.36 | 0.9991 |

**Table S3.** Adsorption Isotherm Coefficients for CH_4_

| **CH4** | **Langmuir model** | | | **Freundlich model** | | | **Langmuir-Freundlich model** | | | |
| --- | --- | --- | --- | --- | --- | --- | --- | --- | --- | --- |
| T (K) | a | b (kPa^-1^) | R^2^ | k | n | R^2^ | a | b (kPa^-1^) | n | R^2^ |
| 273 | 11.35 | 0.0014 | 0.9968 | 1.94 | 5.28 | 0.9938 | 13.55 | 0.0211 | 1.76 | 0.9992 |
| 298 | 11.29 | 0.0008 | 0.9948 | 0.97 | 3.88 | 0.9963 | 12.91 | 0.0162 | 1.52 | 0.9989 |
| 323 | 10.94 | 0.0005 | 0.9980 | 0.42 | 2.95 | 0.9906 | 11.93 | 0.0014 | 1.34 | 0.9986 |
| 348 | 10.86 | 0.0003 | 0.9977 | 0.15 | 2.30 | 0.9957 | 11.12 | 0.0010 | 1.35 | 0.9996 |

**Table S4.** Adsorption Isotherm Coefficients for N_2_

| **N2** | **Langmuir model** | | | **Freundlich model** | | | **Langmuir-Freundlich model** | | | |
| --- | --- | --- | --- | --- | --- | --- | --- | --- | --- | --- |
| T (K) | a | b (kPa^-1^) | R^2^ | k | n | R^2^ | a | b (kPa^-1^) | n | R^2^ |
| 273 | 9.73 | 0.0004 | 0.9993 | 0.30 | 2.77 | 0.9882 | 9.94 | 0.0058 | 1.03 | 0.9994 |
| 298 | 9.62 | 0.0003 | 0.9991 | 0.13 | 2.29 | 0.9893 | 9.45 | 0.0036 | 1.02 | 0.9991 |
| 323 | 9.21 | 0.0001 | 0.9979 | 0.04 | 1.82 | 0.9868 | 8.96 | 0.0034 | 0.88 | 0.9985 |
| 348 | 9.07 | 0.0001 | 0.9978 | 0.02 | 1.65 | 0.9871 | 8.06 | 0.0026 | 0.82 | 0.9992 |

**
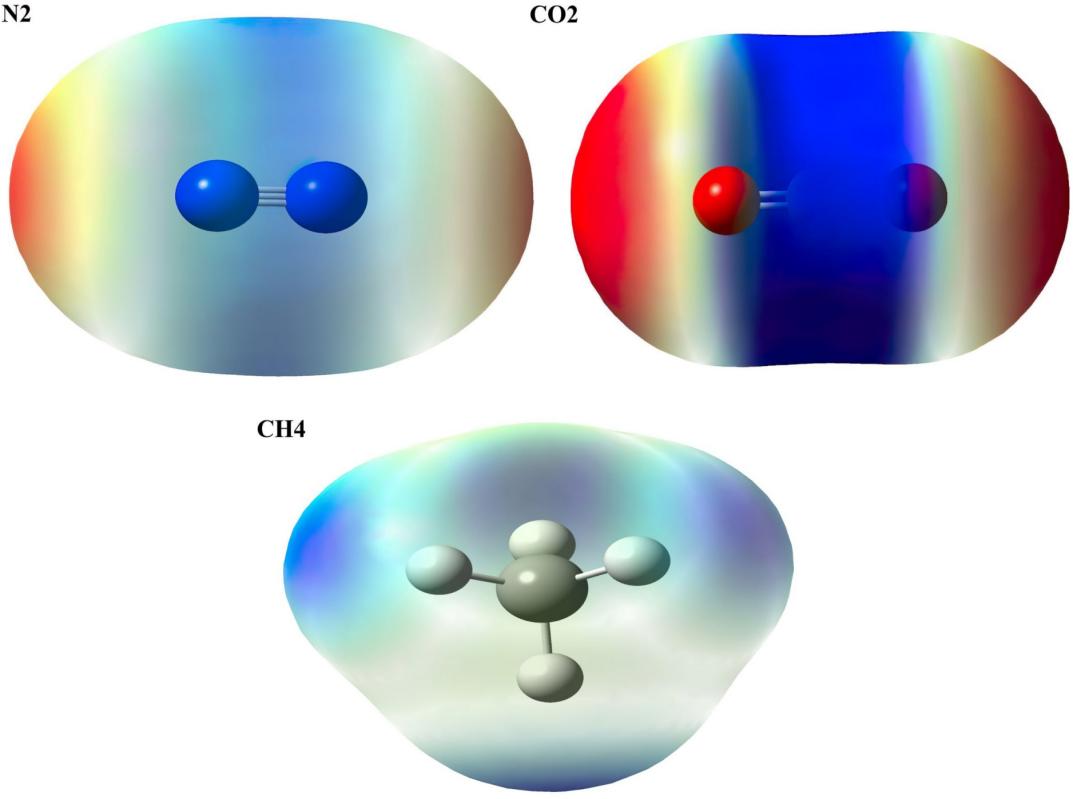
**

**Figure S4.** ESP for N_2_, CO_2_ and CH_4_

**
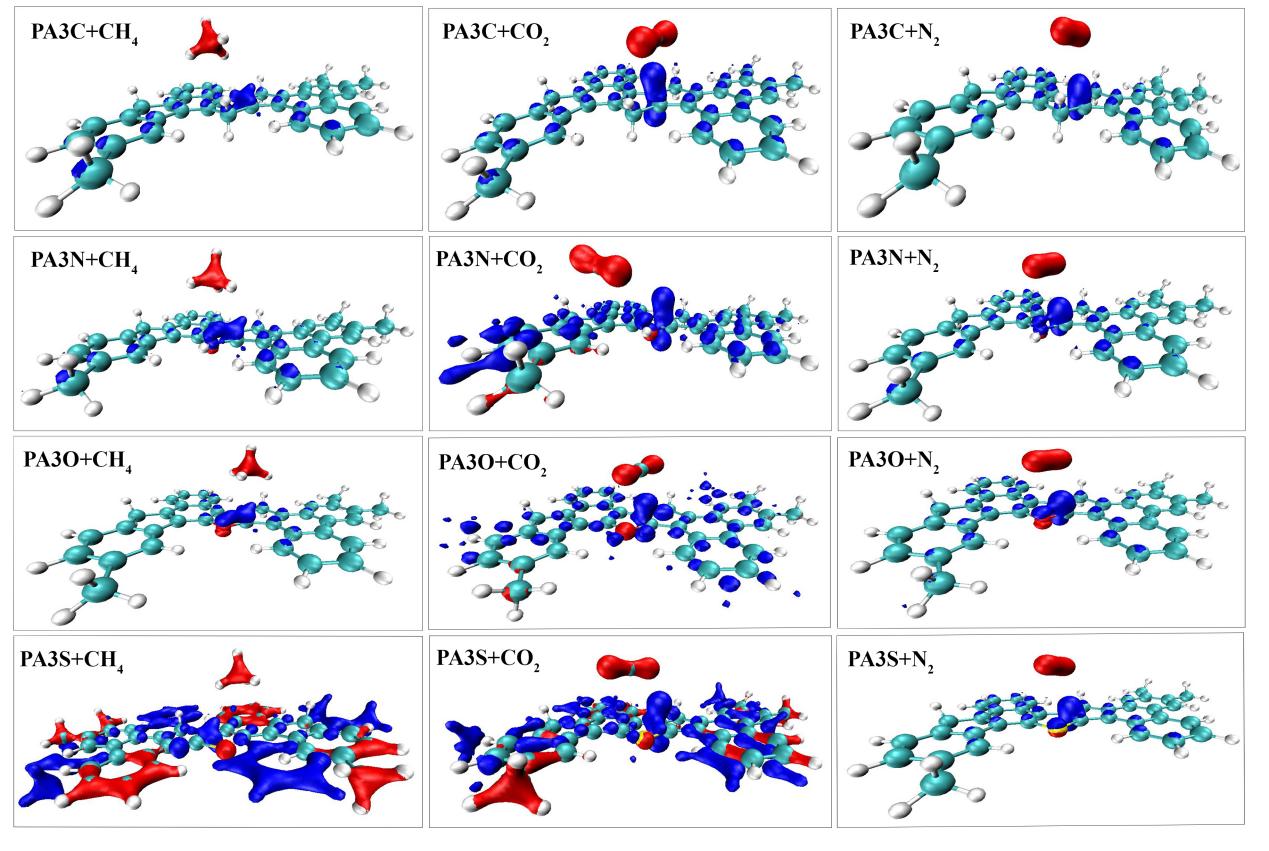
**

**Figure S5.** Electron density difference for adsorption of gas molecules on the surface of asphaltene molecules.


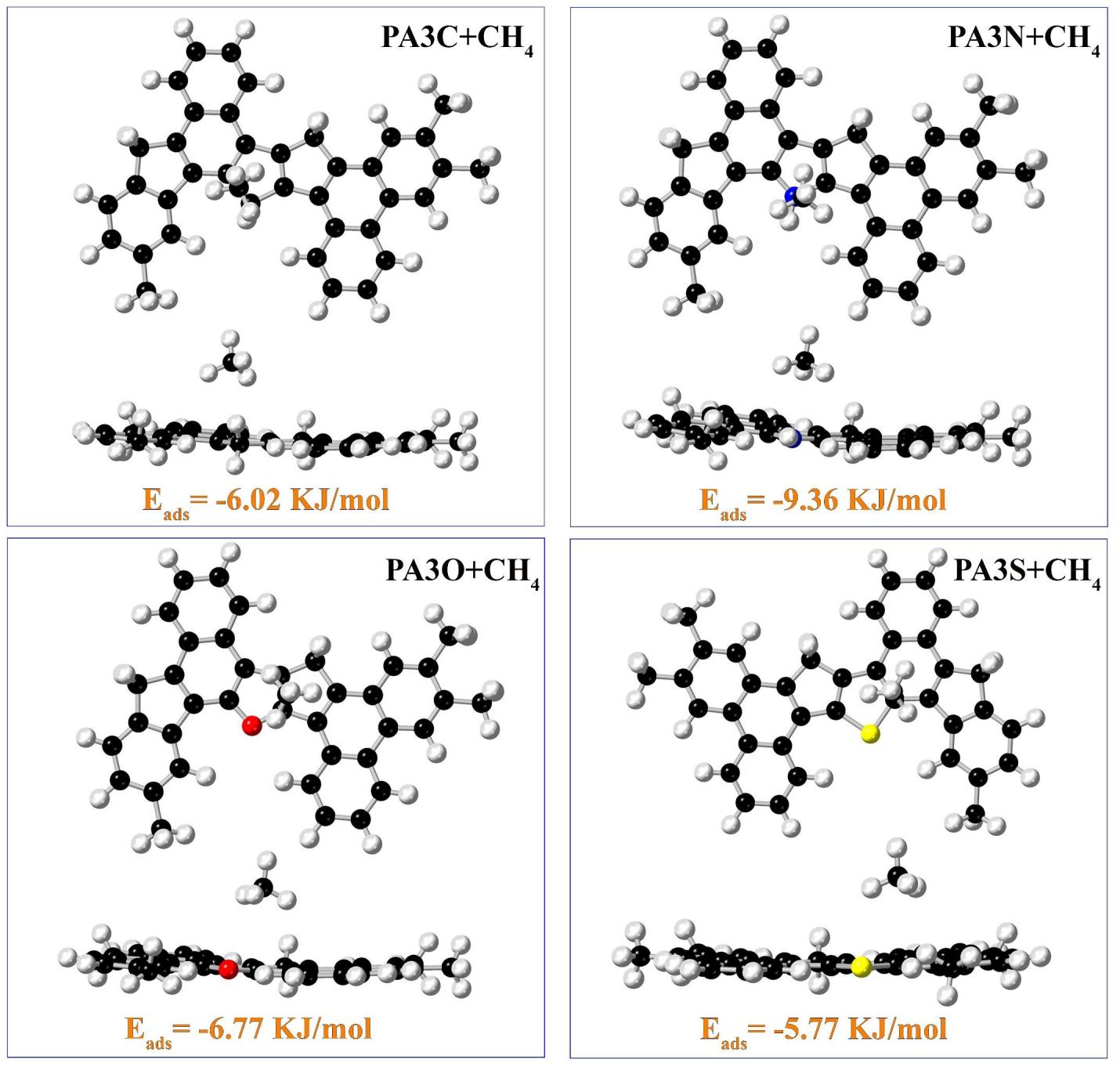


**Figure S6.** Stable adsorption configurations of the CH_4_ molecule adsorbed on the surface of asphaltene fragments .


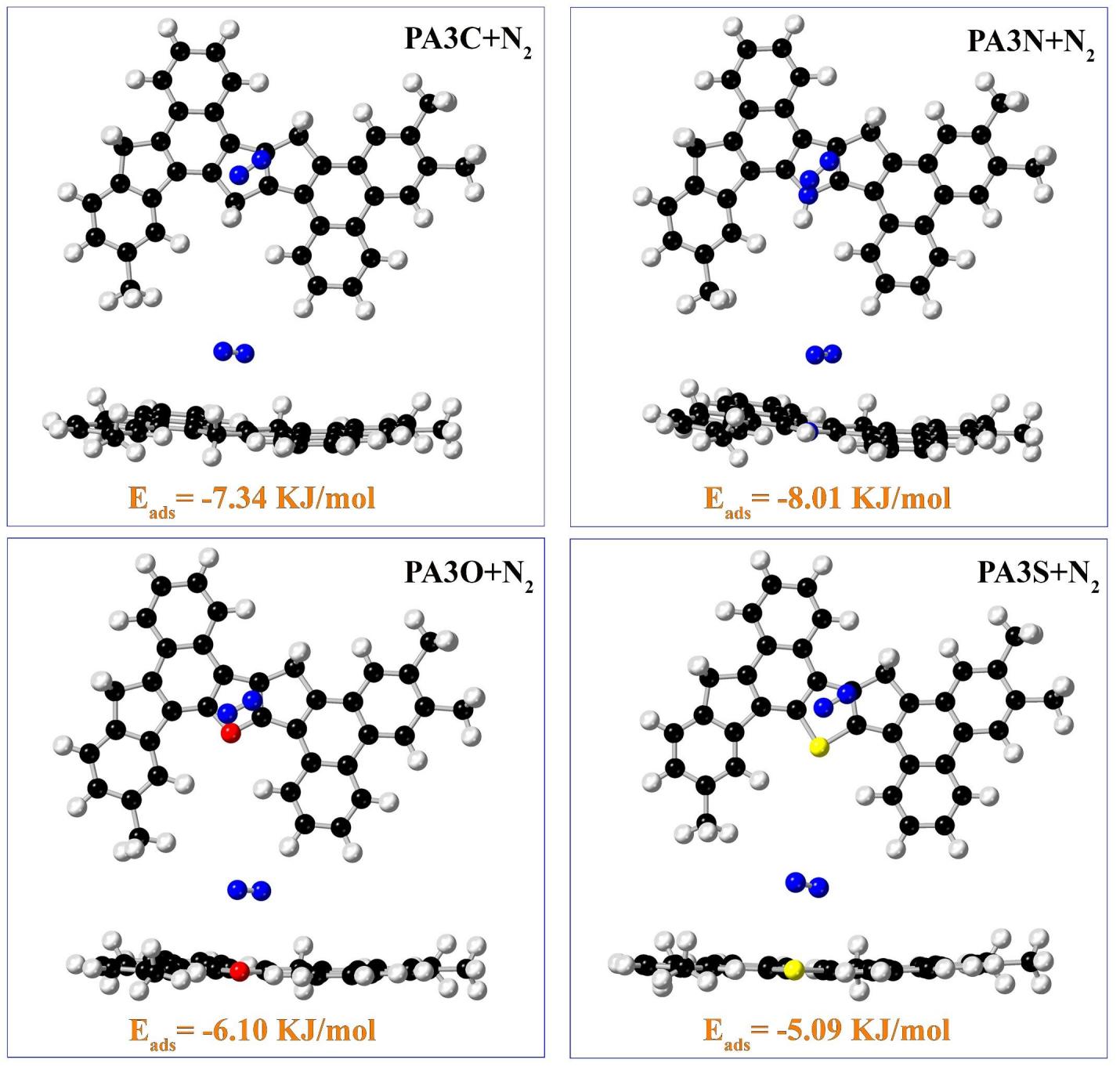


**Figure S7.** Stable adsorption configurations of the N_2_ molecule adsorbed on the surface of asphaltene fragments .

**Table S5.** Adsorption energies

| PA3S | PA3O | PA3N | PA3C | System |
| --- | --- | --- | --- | --- |
| -5.77 KJ/mol | -6.77 KJ/mol | -9.36 KJ/mol | -6.02 KJ/mol | CH_4_ |
| -11.38 KJ/mol | -11.69 KJ/mol | -14.61 KJ/mol | -14.42 KJ/mol | CO_2_ |
| -5.09 KJ/mol | -6.10 KJ/mol | -8.01 KJ/mol | -7.34 KJ/mol | N_2_ |


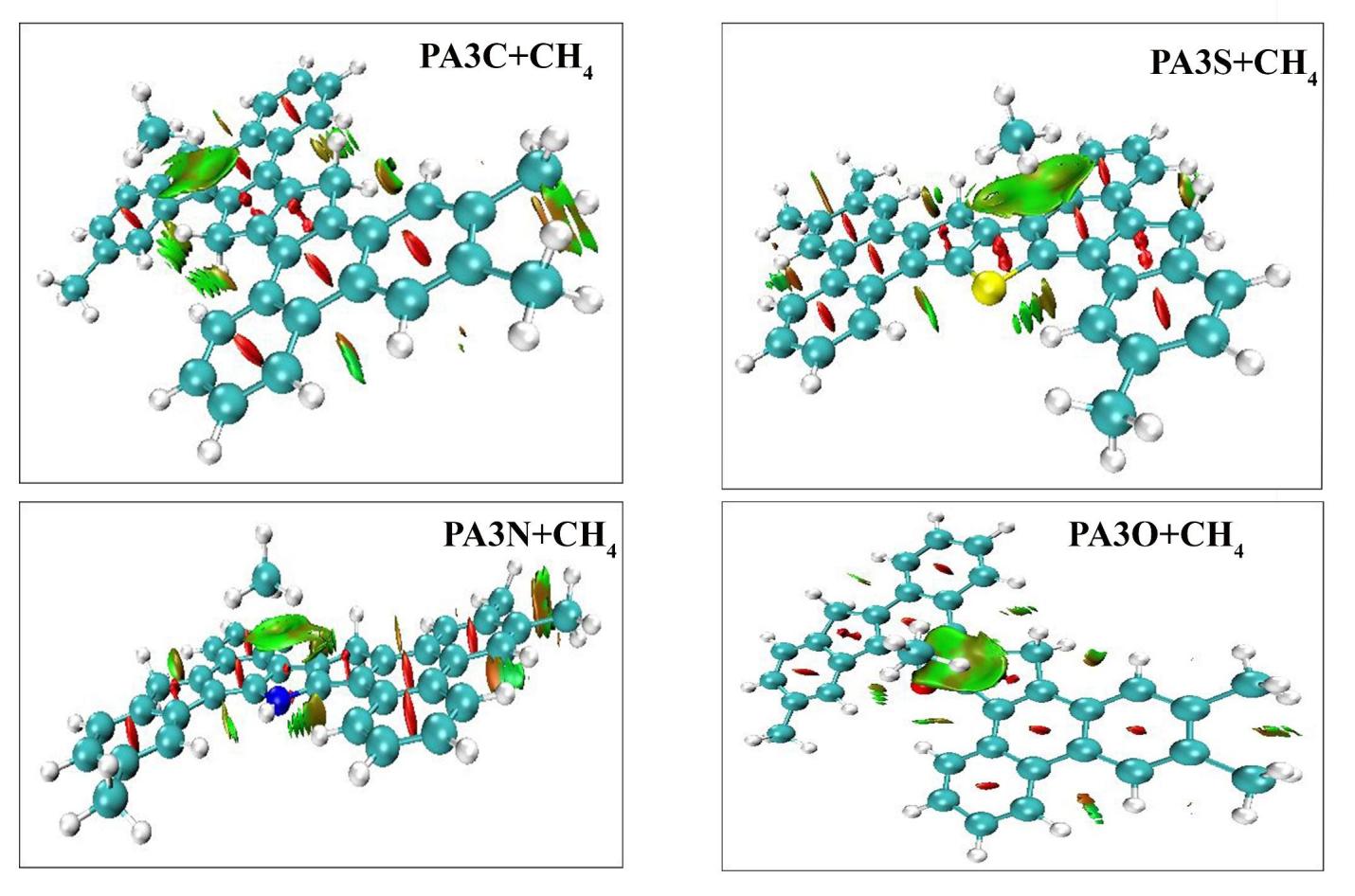


**Figure S8.**Three-dimensional representation of the analysis of non-covalent interactions (NCI) for CH_4_ adsorption on PA3C, PA3S, PA3N and PA3O at the $\omega B97XD$/6-31+g(d,p) level of theory which shows van der Waals interactions. Diagrams were drawn with isosurfaces with RDG 0.6 au. The colors are marked from -0. 05 < (sign λ_2_)ρ < 0.05 au.

**
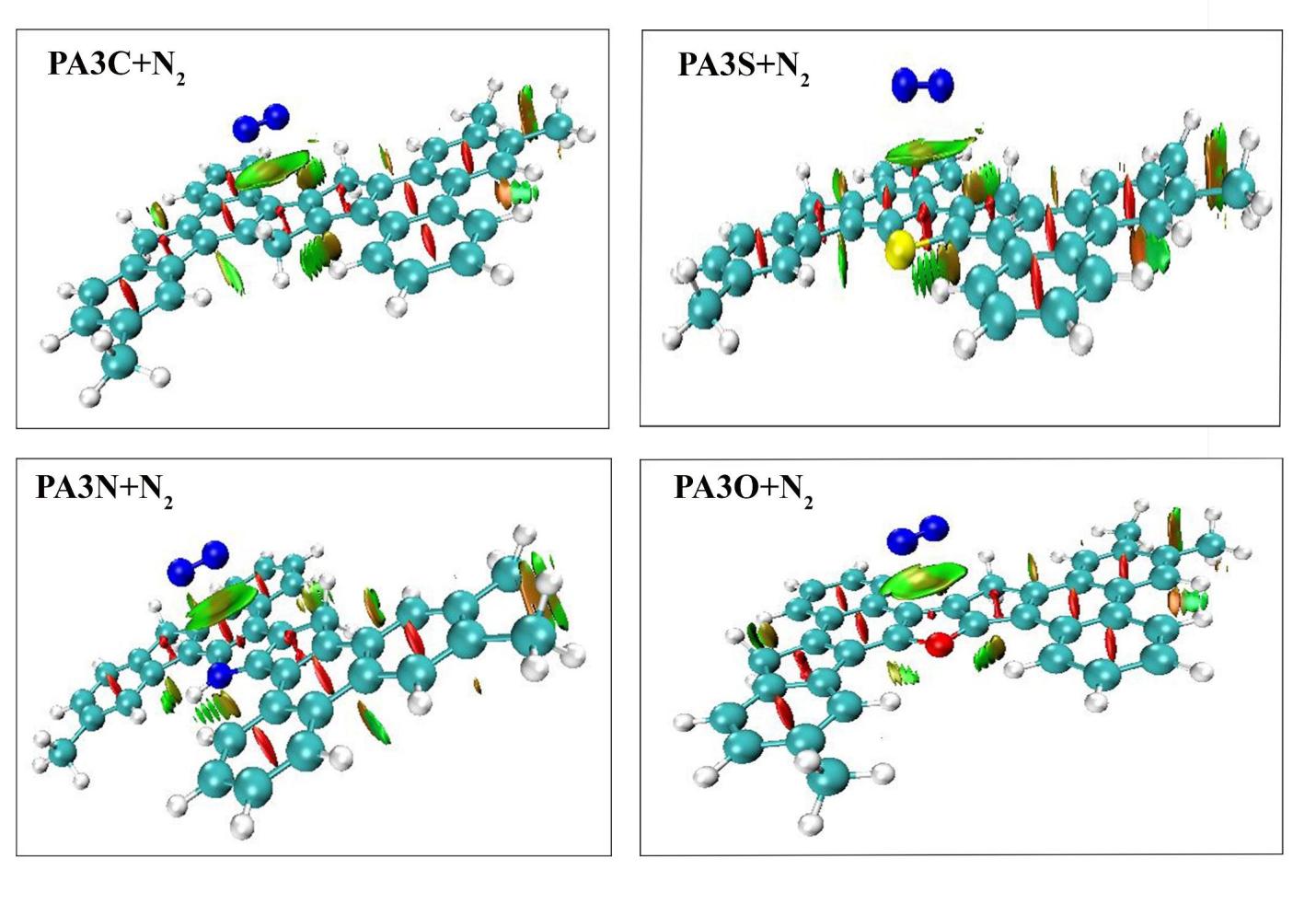
**

**Figure S9.** Three-dimensional representation of the analysis of non-covalent interactions (NCI) for N_2_ adsorption on PA3C, PA3S, PA3N and PA3O at the$\omega B97XD$/6-31+g(d,p) level of theory which shows van der Waals interactions. Diagrams were drawn with isosurfaces with RDG 0.6 au. The colors are marked from -0. 05 < (sign λ_2_)ρ < 0.05 au


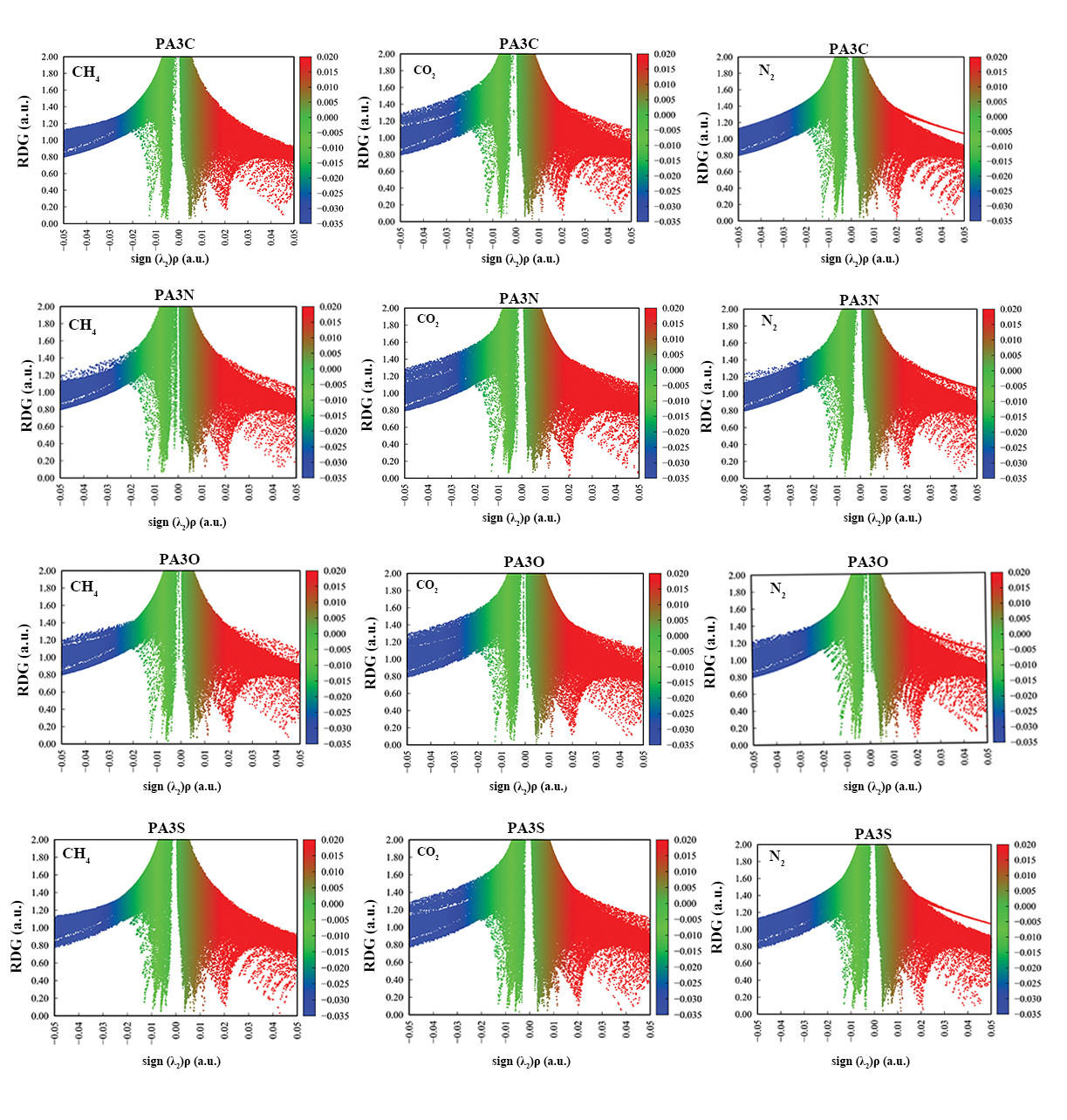


**Figure S10.** Two-dimensional representation of the analysis of non-covalent interactions (NCI) for adsorption of CH_4_, CO_2_, and N_2_ on PA3C, PA3S, PA3N and PA3O at the$\omega B97XD$/6-31+g(d,p) level of theory which shows van der Waals interactions.

**Input**

**PA3C**

| C 5.93202000 1.65634600 0.00160200  C 6.62236000 0.41769700 0.00381200  C 5.90077900 -0.75627800 0.00299700  C 4.49185400 -0.78947700 0.00028900  C 3.81148900 0.44950600 -0.00161200  C 4.55676800 1.64649900 -0.00108000  C 3.73144500 -2.03392800 -0.00037200  C 2.31449600 -1.99444900 -0.00282500  C 1.66374000 -0.71600000 -0.00418400  C 2.38547400 0.44961800 -0.00356400  C 4.35247500 -3.30036600 0.00153400  C 3.61850700 -4.46419800 0.00098800  C 2.21766500 -4.41615700 -0.00157900  C 1.58159000 -3.19824100 -0.00345600  C 0.24962000 -0.36313900 -0.00521700  C 0.11461700 0.98104400 -0.00498700  C 1.45818400 1.64288800 -0.00420600  C -2.02347400 0.15845600 -0.00375200  C -1.29538600 1.34180200 -0.00387100  C -3.42577100 0.20147800 -0.00136200  C -4.07706800 1.42323200 0.00137900  C -3.36524900 2.64556200 0.00105700  C -1.93897000 2.60922400 -0.00194700  C -4.01520700 3.90657000 0.00349600  C -3.29464600 5.07052200 0.00284400  C -1.88306700 5.03355800 -0.00033900  C -1.22491200 3.83242100 -0.00260700  C -4.42798500 -0.87214900 0.00021400  C -5.69898900 -0.28168700 0.00385300  C -5.57131100 1.22096400 0.00466200  C -4.29054300 -2.25465200 -0.00647000  C -5.42794600 -3.06421600 -0.00708100  C -6.68708000 -2.46093900 -0.00619300  C -6.83154400 -1.07492000 0.00006000  C -5.29437700 -4.56551500 0.01788900  C 8.12754700 0.38852100 0.00694400  C 6.69367000 2.95407100 0.00297900  H 6.45716000 -1.68609900 0.00465500  H 4.02284500 2.59214000 -0.00278600  H 5.43237700 -3.37473800 0.00356700  H 4.12771200 -5.42138500 0.00258500  H 1.64148600 -5.33453100 -0.00199700  H 0.49892800 -3.15643000 -0.00543800  H -5.09955500 3.94097300 0.00589200  H -3.80637600 6.02644100 0.00471600  H -1.32195600 5.96129800 -0.00098500  H -0.14127300 3.81136500 -0.00514500  H -6.04266400 1.66731000 0.88754100  H -3.30823800 -2.71503500 -0.01373700  H -7.57313000 -3.08824400 -0.01266000  H -7.82175900 -0.63080600 -0.00209700  H -6.17165900 -5.04994800 -0.41603000  H -5.18842600 -4.92996300 1.04470600  H -4.41462500 -4.89451600 -0.54031100  H 8.50108800 -0.63685900 0.01019500  H 8.53483200 0.89749800 0.88633600  H 8.53838600 0.89319800 -0.87329200  H 7.33274400 3.03869000 0.88755200  H 6.01386400 3.80774000 -0.00483400  H 7.34652100 3.03300900 -0.87193100  H 1.61136600 2.27436200 0.87836500  H 1.61250200 2.27441900 -0.88654800  H -6.04660400 1.66855900 -0.87544600  C -1.09136000 -1.03104200 -0.00513200  H -1.24578700 -1.66297800 0.87716400  H -1.24625900 -1.66122200 -0.88861400 |
| --- |

**Input**

**PA3N**

| C 5.94392900 1.62319800 0.00495500  C 6.60335900 0.36763800 0.01153600  C 5.85285900 -0.78769300 0.00937800  C 4.44354100 -0.78481400 0.00148100  C 3.79387200 0.46997600 -0.00409500  C 4.56903100 1.64819800 -0.00275000  C 3.65302400 -2.00916700 -0.00082700  C 2.23724400 -1.93498800 -0.00744500  C 1.62055700 -0.64055400 -0.01002000  C 2.36798400 0.50986500 -0.00927000  C 4.24429000 -3.28977800 0.00298700  C 3.48558400 -4.43732900 -0.00000700  C 2.08669600 -4.35694500 -0.00752600  C 1.48085800 -3.12406800 -0.01115800  C 0.22446000 -0.22829500 -0.01151100  C 0.11613200 1.13684200 -0.01208400  C 1.48531500 1.74231900 -0.01149200  C -1.95092600 0.23079300 -0.00799400  C -1.26857500 1.45987600 -0.00961800  C -3.36148800 0.17569300 -0.00249300  C -4.06335200 1.36075100 0.00296800  C -3.42073000 2.63273300 0.00090300  C -1.99911500 2.68791900 -0.00625000  C -4.14831600 3.84516900 0.00557300  C -3.50422500 5.05745800 0.00299200  C -2.09780300 5.10908300 -0.00451800  C -1.36396700 3.94769800 -0.00893900  C -4.30544300 -0.94830400 0.00121400  C -5.60493800 -0.41743900 0.01074500  C -5.54543500 1.08782500 0.01168500  C -4.11262700 -2.32340400 -0.00609500  C -5.21339200 -3.18452100 -0.00112900  C -6.49636500 -2.63902900 0.00798700  C -6.69962100 -1.25914900 0.01394700  C -5.00406800 -4.67726700 0.00767500  C 8.10719000 0.30076500 0.02060000  C 6.73829700 2.90105300 0.00826300  H 6.38615700 -1.73080700 0.01444600  H 4.05894200 2.60657400 -0.00772300  H 5.32208300 -3.38829300 0.00835600  H 3.97314800 -5.40553500 0.00313700  H 1.48818200 -5.26078000 -0.01062200  H 0.39922100 -3.06829300 -0.01825500  H -5.23278400 3.81043000 0.01130300  H -4.07717200 5.97796400 0.00657500  H -1.59406400 6.06926500 -0.00683200  H -0.28011700 3.99104400 -0.01491300  H -6.03365700 1.51164700 0.89647100  H -3.11724300 -2.75704800 -0.01794800  H -7.35375700 -3.30451600 0.00900100  H -7.70790100 -0.85769800 0.01914700  H -5.94305900 -5.21005900 -0.15333000  H -4.59189600 -5.00902500 0.96559100  H -4.30445300 -4.98295400 -0.77503900  H 8.45476200 -0.73368800 0.02848600  H 8.52356400 0.80233000 0.89992400  H 8.53360600 0.79227600 -0.85962900  H 7.37038000 2.97274800 0.89902200  H 6.08107200 3.77201000 -0.00977100  H 7.40192800 2.95898200 -0.86009800  H 1.67361100 2.36448200 0.87073000  H 1.67557100 2.36268800 -0.89458700  H -6.04427800 1.51268400 -0.86666000  N -1.02539400 -0.79147200 -0.01032600  H -1.24709400 -1.76878100 -0.00248700 |
| --- |

**Input**

**PA3O**

C 5.96548600 1.60033500 -0.00171200

C 6.58591300 0.32243300 0.00017100

C 5.79761900 -0.81187100 0.00107300

C 4.38709500 -0.76560700 0.00022500

C 3.77649000 0.51345800 -0.00166900

C 4.58852000 1.66870500 -0.00256800

C 3.56087400 -1.97020100 0.00137700

C 2.14695600 -1.85476900 -0.00005200

C 1.57747100 -0.54039900 -0.00218700

C 2.35024000 0.59621800 -0.00249000

C 4.10954100 -3.27222300 0.00431700

C 3.30528900 -4.39307500 0.00574900

C 1.90615000 -4.26517700 0.00431700

C 1.33712500 -3.01088800 0.00141600

C 0.20343700 -0.08013100 -0.00301000

C 0.10763400 1.27355200 -0.00308900

C 1.49084800 1.85287600 -0.00314300

C -1.91293900 0.31841800 -0.00158000

C -1.29401100 1.56724300 -0.00175500

C -3.30659000 0.16264800 0.00018800

C -4.08165800 1.30781900 0.00333500

C -3.51043600 2.61386600 0.00331500

C -2.08926800 2.75344400 0.00017800

C -4.30410100 3.78911900 0.00563600

C -3.72449200 5.03611500 0.00472800

C -2.31900300 5.16798000 0.00145300

C -1.52046700 4.04759400 -0.00068700

C -4.16910400 -1.02040200 0.00006700

C -5.50122300 -0.57804500 0.00381800

C -5.54576400 0.93242800 0.00604800

C -3.85883400 -2.37609400 -0.00949600

C -4.89394400 -3.31611600 -0.01253500

C -6.21981300 -2.86557100 -0.01046500

C -6.53221600 -1.50415900 -0.00155500

C -4.58442100 -4.79291300 0.00759800

C 8.08849200 0.20790300 0.00119800

C 6.80054400 2.85403700 -0.00285800

H 6.30126600 -1.77265800 0.00247100

H 4.10777700 2.64385500 -0.00407700

H 5.18450300 -3.41029800 0.00573600

H 3.75944800 -5.37895500 0.00824200

H 1.27783200 -5.15023300 0.00571300

H 0.25778800 -2.89595900 0.00063600

H -5.38626900 3.69501100 0.00809600

H -4.34848800 5.92444500 0.00639700

H -1.86912900 6.15575000 0.00056800

H -0.43863600 4.14713000 -0.00331600

H -6.06496700 1.32286900 0.89016700

H -2.82228300 -2.70089800 -0.01728900

H -7.02477900 -3.59587700 -0.01854400

H -7.57064000 -1.18404100 -0.00336800

H -5.37280900 -5.37183800 -0.48149800

H -4.49390200 -5.15912100 1.03641500

H -3.64071300 -5.00612600 -0.50223800

H 8.40357200 -0.83839400 0.00186200

H 8.52598100 0.69099500 0.88210600

H 8.52714800 0.69026500 -0.87951900

H 7.45133800 2.90032800 0.87748600

H 6.17071800 3.74698200 -0.00330900

H 7.45070900 2.89904300 -0.88375300

H 1.69015600 2.47065800 0.88075500

H 1.69032500 2.47009000 -0.88740400

H -6.06875200 1.32502800 -0.87485300

O -1.00022900 -0.70366900 -0.00245300

**Input**

**PA3S**

C 5.94763200 1.73242400 -0.00043300

C 6.68086800 0.51586600 -0.00174100

C 5.99852100 -0.68518100 -0.00212100

C 4.58947000 -0.76626800 -0.00133300

C 3.86710100 0.45081300 -0.00012800

C 4.57007400 1.67491100 0.00035300

C 3.87004200 -2.03699100 -0.00174400

C 2.44935900 -2.04815900 -0.00103800

C 1.75921100 -0.78787900 -0.00009100

C 2.43937300 0.40294100 0.00041500

C 4.53780800 -3.28161900 -0.00283500

C 3.84577200 -4.47439400 -0.00313000

C 2.44196100 -4.47650600 -0.00229800

C 1.76028500 -3.27961000 -0.00128300

C 0.33492400 -0.46602900 0.00025900

C 0.14251100 0.88805100 0.00097300

C 1.48189000 1.57148200 0.00129000

S -1.14885900 -1.35491100 -0.00037800

C -2.06198800 0.13506900 0.00016200

C -1.23738100 1.26921300 0.00098700

C -3.47162600 0.25598800 -0.00019200

C -4.03723000 1.51394100 0.00031400

C -3.24723700 2.69808400 0.00140400

C -1.82692000 2.58058100 0.00173100

C -3.83160400 3.98877900 0.00201500

C -3.05078000 5.12052100 0.00304200

C -1.64590900 5.00578600 0.00345300

C -1.05126900 3.76445800 0.00279600

C -4.54034200 -0.75368700 -0.00039900

C -5.77130400 -0.07784600 -0.00081400

C -5.54255200 1.41304800 -0.00056000

C -4.49644200 -2.14502500 -0.00482500

C -5.68915000 -2.87615700 -0.00709800

C -6.90742400 -2.18855600 -0.01058500

C -6.95752100 -0.79297300 -0.00667600

C -5.65702400 -4.38451500 0.02320700

C 8.18775500 0.53727400 -0.00273100

C 6.66436900 3.05726900 -0.00007000

H 6.58717600 -1.59642600 -0.00309500

H 4.00359300 2.60302500 0.00132500

H 5.62097900 -3.31593500 -0.00346800

H 4.39001300 -5.41354600 -0.00398400

H 1.89684800 -5.41489500 -0.00243700

H 0.67529400 -3.28593700 -0.00056000

H -4.91397300 4.07862500 0.00168800

H -3.51433300 6.10191100 0.00354000

H -1.03079400 5.90024700 0.00426600

H 0.03015700 3.69436200 0.00313800

H -5.98477500 1.89214700 0.88183600

H -3.54885700 -2.67611800 -0.00813000

H -7.83529900 -2.75457200 -0.01820900

H -7.91628100 -0.28181500 -0.01195400

H -6.55425700 -4.81174800 -0.43287100

H -5.60192400 -4.75227100 1.05395500

H -4.78623400 -4.77396700 -0.51165100

H 8.59661700 -0.47599100 -0.00393400

H 8.58131900 1.05647900 0.87834300

H 8.58012300 1.05805700 -0.88340700

H 7.30866200 3.16228300 0.87989500

H 5.95414900 3.88754000 0.00182300

H 7.30598600 3.16411300 -0.88177800

H 1.62497900 2.20436100 0.88616000

H 1.62479600 2.20563700 -0.88268600

H -5.98356100 1.89239700 -0.88340300

Refernce:

1. Ekramipooya, A., et al., *Effect of the heteroatom presence in different positions of the model asphaltene structure on the self-aggregation: MD and DFT study.* Journal of Molecular Liquids, 2021. **334**: p. 116109.

2. Hu, H., et al., *Small-molecule gas sorption and diffusion in coal: Molecular simulation.* Energy, 2010. **35**(7): p. 2939-2944.
